# GC-MS-Based Metabolic Profiling Identifies Candidate Endogenous Inhibitors Associated with the Physiological Component of Morphophysiological Dormancy in *Spuriopinella brachycarpa* (Kom.) Kitag. Seeds

**DOI:** 10.3390/plants15213274

**Published:** 2026-10-23

**Authors:** Shanshan Chen, Yan Zou, Ze Li, Shuang Liu, Jianlei Qiao, Yue Yu, Xin Cao, Jing Zhao, Shuyao Song, Yue Zou, Jiayu Teng, Huixin Lv, Qin Qi, Jiaming Li, Chunlei Yu, Chengxu Wang, Baiming Yang

**Affiliations:** 1 College of Horticulture, Jilin Agricultural University, Changchun 130118, China; 2 Faculty of Agriculture, Jilin Agricultural Science and Technology College, Changchun 130118, China

**Keywords:** *Spuriopinella brachycarpa* (Kom.) Kitag., morphophysiological dormancy, endogenous germination inhibitors, GC-MS, seed dormancy

## Abstract

Seeds of *Spuriopinella brachycarpa* (Kom.) Kitag., an economically valuable wild vegetable of the Apiaceae family, exhibit morphophysiological dormancy (MPD), which is characterized by underdeveloped embryos at dispersal, together with a physiological dormancy component that requires specific environmental cues for release. However, the endogenous chemical basis of this physiological component remains unclear. In this study, compounds associated with inhibitory activity were extracted from freshly harvested seeds using organic solvents of graded polarity (methanol, diethyl ether, petroleum ether, and ethyl acetate), and the inhibitory activity of each fraction was evaluated in heterologous bioassays using Chinese cabbage (*Brassica rapa* subsp. *pekinensis*) seeds. The methanol-phase extract exhibited the strongest inhibitory effect, reducing the germination percentage by 83.40% relative to the water control, followed by the diethyl ether extract (45.34% reduction), whereas the petroleum ether and ethyl acetate extracts showed only weak effects. GC-MS profiling of the extracts identified 65 compounds, predominantly organic acids, ketones, phenols, esters, and monoterpenes. Based on their relative abundance and previously reported germination inhibitory activities, acetic acid, palmitic acid, benzaldehyde, and thymol were tentatively identified priority candidate compounds associated with inhibitory activity. All compound identities are putative assignments based on NIST library spectral matching (match score > 80%), and their associations with inhibitory activity are correlative rather than causal. These findings provide the first systematic chemical characterization of compounds associated with inhibitory activity in *S. brachycarpa* seeds and a biochemical basis for understanding the physiological component of MPD, while establishing practical candidate targets for developing seed priming formulations and dormancy-breaking techniques. Further verification using authentic standards and direct bioassays on *S. brachycarpa* embryos is required to confirm the causal roles of these compounds.

## 1. Introduction

*Spuriopinella brachycarpa* (Kom.) Kitag., alias pseudo-anise, dimethyl anise, short-fruited anise, etc., is a perennial wild herb of the family Apiaceae [1]. As a special wild vegetable in the Changbai Mountain area, *S. brachycarpa* is delicious and rich in various nutrients, including proteins, vitamins, mineral elements, and crude fiber and has high edible value. Meanwhile, it has been traditionally used for assisting in lowering blood pressure, enhancing appetite, and blood supplementation, indicating its potential medicinal value and broad economic prospects. In recent years, scholars and experts have conducted extensive research on *S. brachycarpa*, including seed germination technology [2], intercropping cultivation techniques [3], tissue culture and rapid propagation [4], as well as the development and utilization of wild vegetable resources [5]. Previous studies showed that the seeds of *S. brachycarpa* exhibit morphophysiological dormancy (MPD), a complex dormancy type involving both underdeveloped embryos at dispersal and a physiological component requiring specific environmental cues for dormancy release [3]. According to Baskin and Baskin [6], seeds of the Apiaceae family typically exhibit MPD, which involves two distinct processes: embryo growth and differentiation (morphological component), and physiological dormancy release, often requiring warm stratification followed by cold stratification and/or gibberellic acid (GA_3_) application. The causes of seed dormancy vary among plant species, and physiological dormancy caused by compounds associated with inhibitory activity is a major contributing factor [7]. Germination inhibitors primarily suppress radicle elongation, thereby inducing seed dormancy [8]. To identify the specific types of compounds associated with inhibitory activity responsible for dormancy in *S. brachycarpa* seeds, this study examined the role of compounds associated with inhibitory activity in the dormancy of freshly harvested seeds, aiming to provide a theoretical basis for understanding the physiological mechanisms of dormancy and germination in this species.

It is important to distinguish that MPD comprises two independent components: the morphological component (embryo underdevelopment) and the physiological component (internal physiological state involving hormonal regulation, notably ABA dominance). While endogenous chemical inhibitors may strengthen the physiological component of MPD, they cannot be considered the primary cause of the morphological component (i.e., underdeveloped embryos). Furthermore, physiological dormancy (PD) is distinct from chemical dormancy, a classification that has been rejected in modern seed dormancy taxonomy [9]. Therefore, the present study aims to identify endogenous compounds that may contribute to the physiological component of MPD in *S. brachycarpa*, rather than claiming these inhibitors as the sole cause of seed dormancy. This investigation addresses a critical practical bottleneck: the poor and erratic germination that has hindered large-scale commercial cultivation of this economically valuable species. While metabolic profiling provides a snapshot of endogenous chemical composition, transcriptomic analysis of dormancy release dynamics is essential to understand the regulatory networks governing these metabolic changes. Complementary GC-MS-based chemical profiling of freshly harvested seeds and RNA-Seq characterization of stratification-induced dormancy release were conducted in parallel; however, as the two datasets were derived from different sample sets, they provide contextual rather than directly correlated insights. Morphological dormancy is caused by an underdeveloped embryo, whereas physiological dormancy involves physiological blocks in a fully developed embryo. Morphophysiological dormancy combines both. Chemical inhibition, by contrast, refers to germination suppression by endogenous compounds and may co-occur with physiological dormancy but should be conceptually distinguished from it.

Because germination inhibitors are characteristically lipophilic or semi-volatile small molecules (e.g., fatty acids, phenolics, terpenoids), direct-injection GC-MS was selected as the primary screening platform. We acknowledge that this approach does not capture polar metabolites; LC-MS and derivatization-based workflows are tentatively identified necessary complements in future work. To characterize the chemical basis of the physiological component of MPD, we profiled endogenous compounds in freshly harvested seeds by direct-injection GC-MS and evaluated inhibitory activity through bioassays. In parallel, we performed RNA-Seq at three stratification stages (0, 60, and 120 days) selected to represent dormancy maintenance, transitional release, and full germination, respectively.

## 2. Results

### 2.1. Determination of Biological Activity of Organic-Phase Extracts from S. brachycarpa Seeds

The effects of different organic-phase extracts from *S. brachycarpa* seeds on the germination and growth of Chinese cabbage seeds are shown in Table 1. Both methanol-phase and ether-phase extracts exhibited inhibitory effects on seed germination. The methanol-phase extract showed the most pronounced inhibition, which was significantly different from the control (*p* < 0.05). In contrast, the inhibitory effects of petroleum ether-phase and ethyl acetate-phase extracts were relatively weak, showing no significant difference compared to the control.

The effects of the organic-phase extracts of *S. brachycarpa* seeds on the germination and growth of Chinese cabbage seeds are shown in Table 1. The methanol- and diethyl ether-phase extracts significantly inhibited seed germination compared with the water control (*p* < 0.05), whereas the inhibitory effects of the petroleum ether- and ethyl acetate-phase extracts were weak and not significantly different from the control. The germination percentages of Chinese cabbage seeds treated with the methanol-, diethyl ether-, petroleum ether-, and ethyl acetate-phase extracts were 13.67 ± 1.53%, 45.00 ± 1.00%, 79.33 ± 2.31%, and 78.00 ± 3.61%, respectively, corresponding to 16.60%, 54.66%, 96.36%, and 94.74% of the control value (82.33 ± 2.08%), that is, reductions of 83.40%, 45.34%, 3.64%, and 5.26%. The methanol-phase extract also caused the greatest reductions in root length (2.23 cm) and seedling height (2.33 cm), followed by the diethyl ether extract (1.77 cm and 1.66 cm, respectively), while the petroleum ether and ethyl acetate extracts showed only weak effects on seedling growth (Table 1).

In summary, among the four organic-solvent extracts tested, the methanol-phase extract demonstrated the strongest inhibitory effect on the germination and growth of Chinese cabbage seeds, followed by the diethyl ether-phase extract. These results suggest that the endogenous inhibitory substances of *S. brachycarpa* seeds are readily soluble in polar solvents such as methanol.

### 2.2. Types of Organic Compounds Contained in Organic-Phase Extracts of S. brachycarpa Seeds

**Figure 1:**
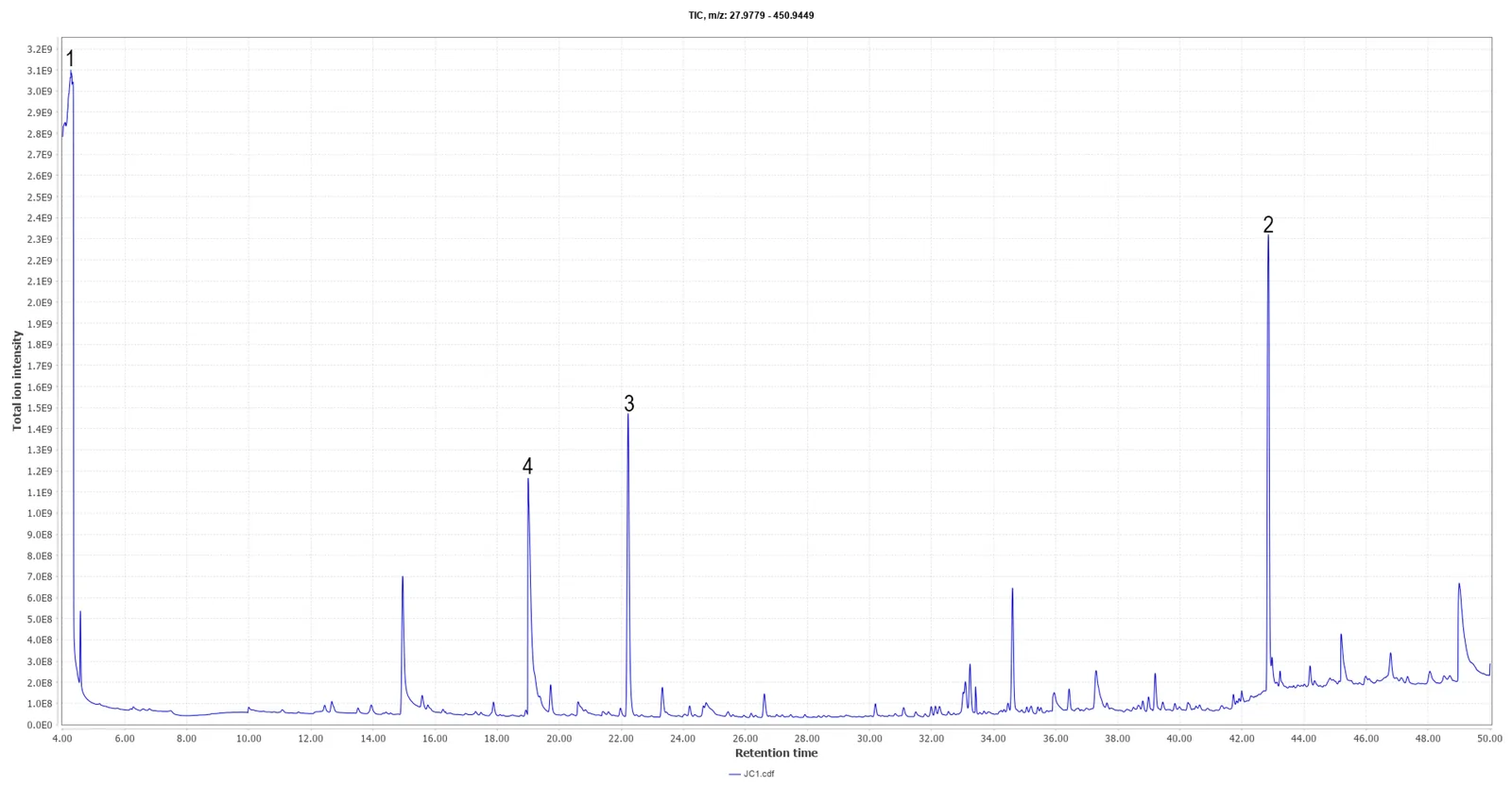
Total ion chromatogram (TIC) of the methanol-phase extract of *Spuriopinella brachycarpa* (Kom.) Kitag. seeds. Numbered peaks correspond to 1, ethanol (4.39 min); 2, ethylbenzene (9.17 min); 3, n-methylpyrrole (9.66 min); 4, acetic acid (19.01 min).

**Figure 2:**
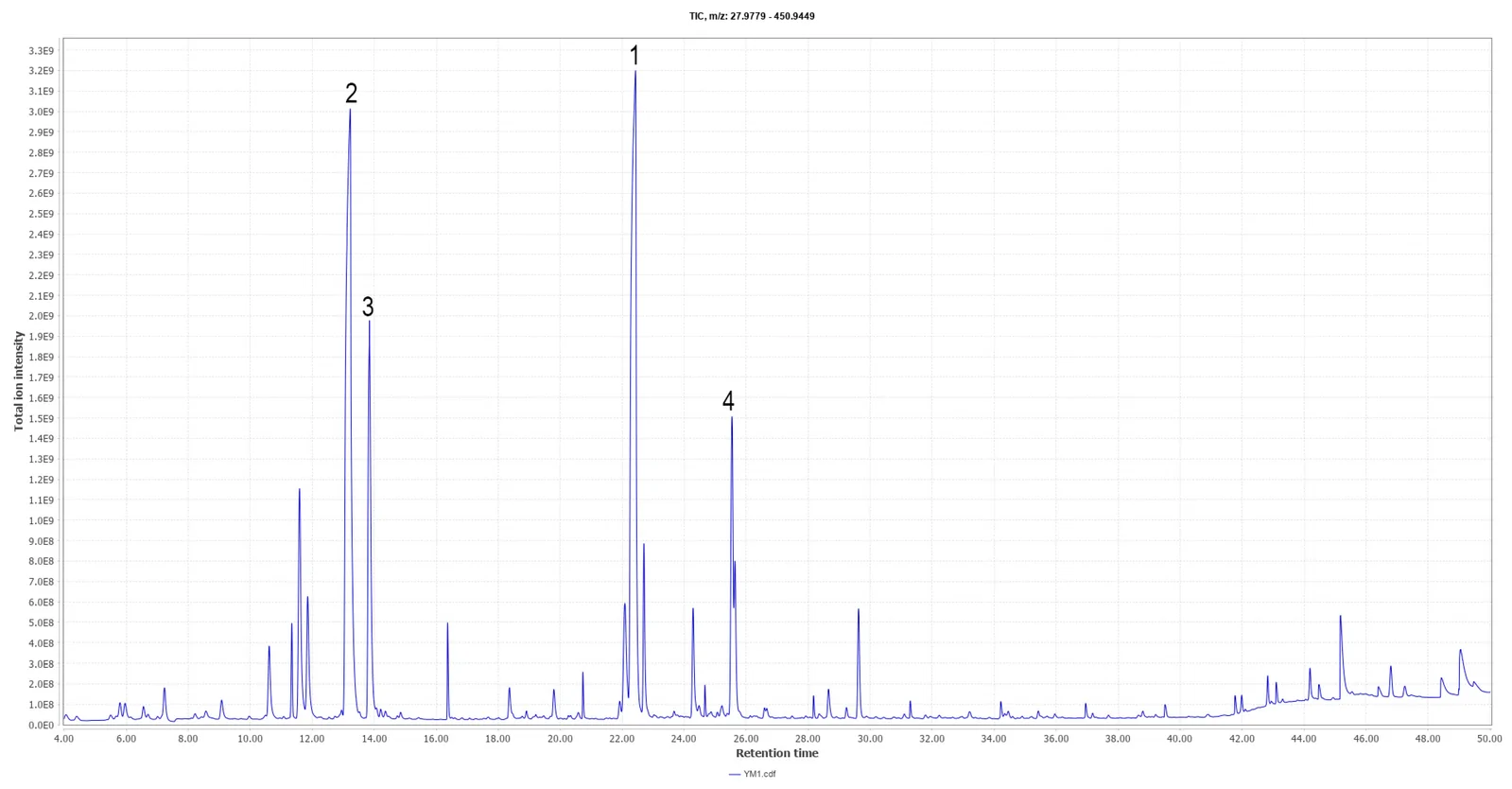
Total ion chromatogram (TIC) of ether phase of *Spuriopinella brachycarpa* (Kom.) Kitag. seeds.

**Figure 3:**
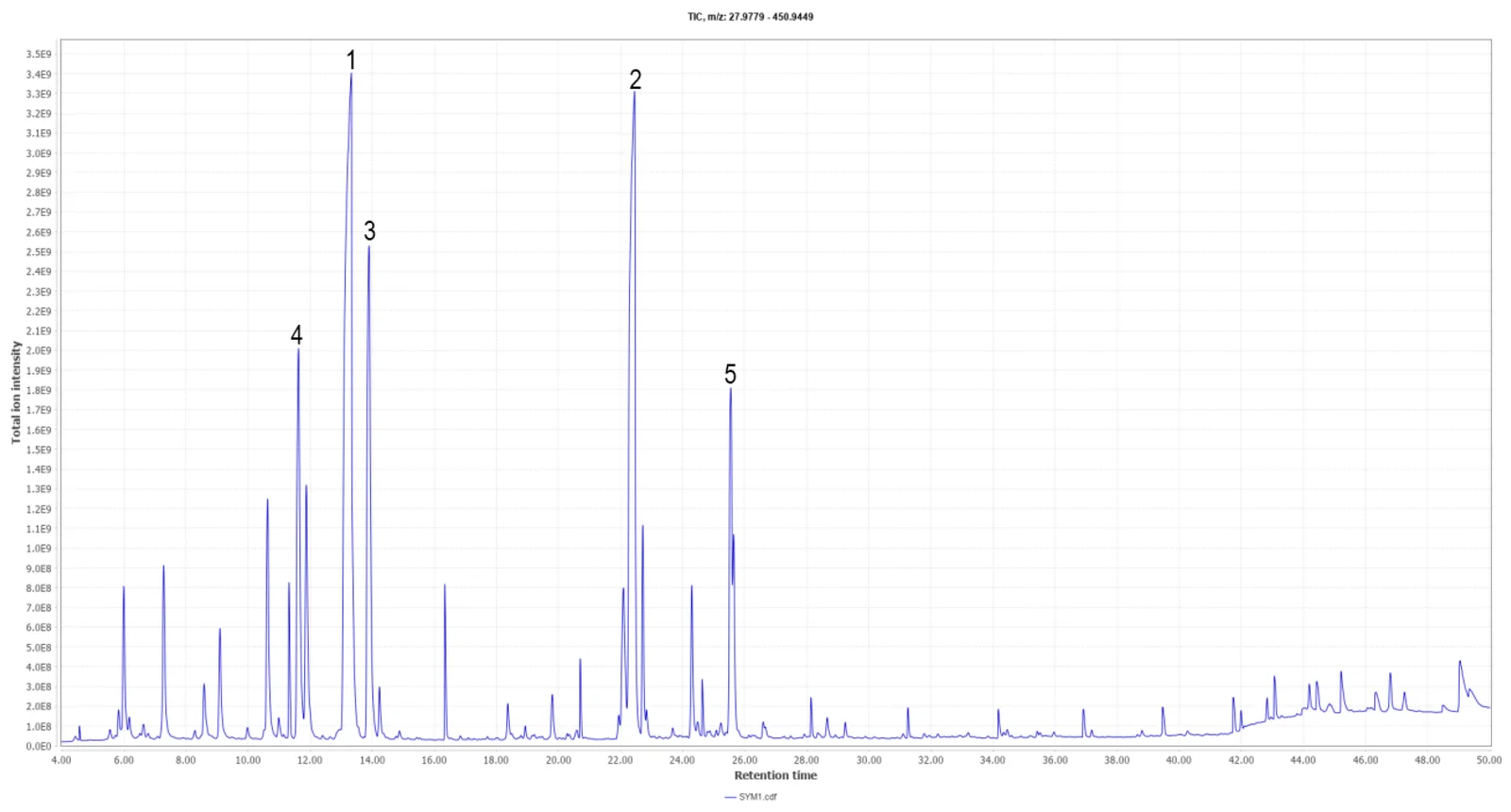
Total ion chromatogram (TIC) of petroleum ether extract of *Spuriopinella brachycarpa* (Kom.) Kitag. seeds.

**Figure 4:**
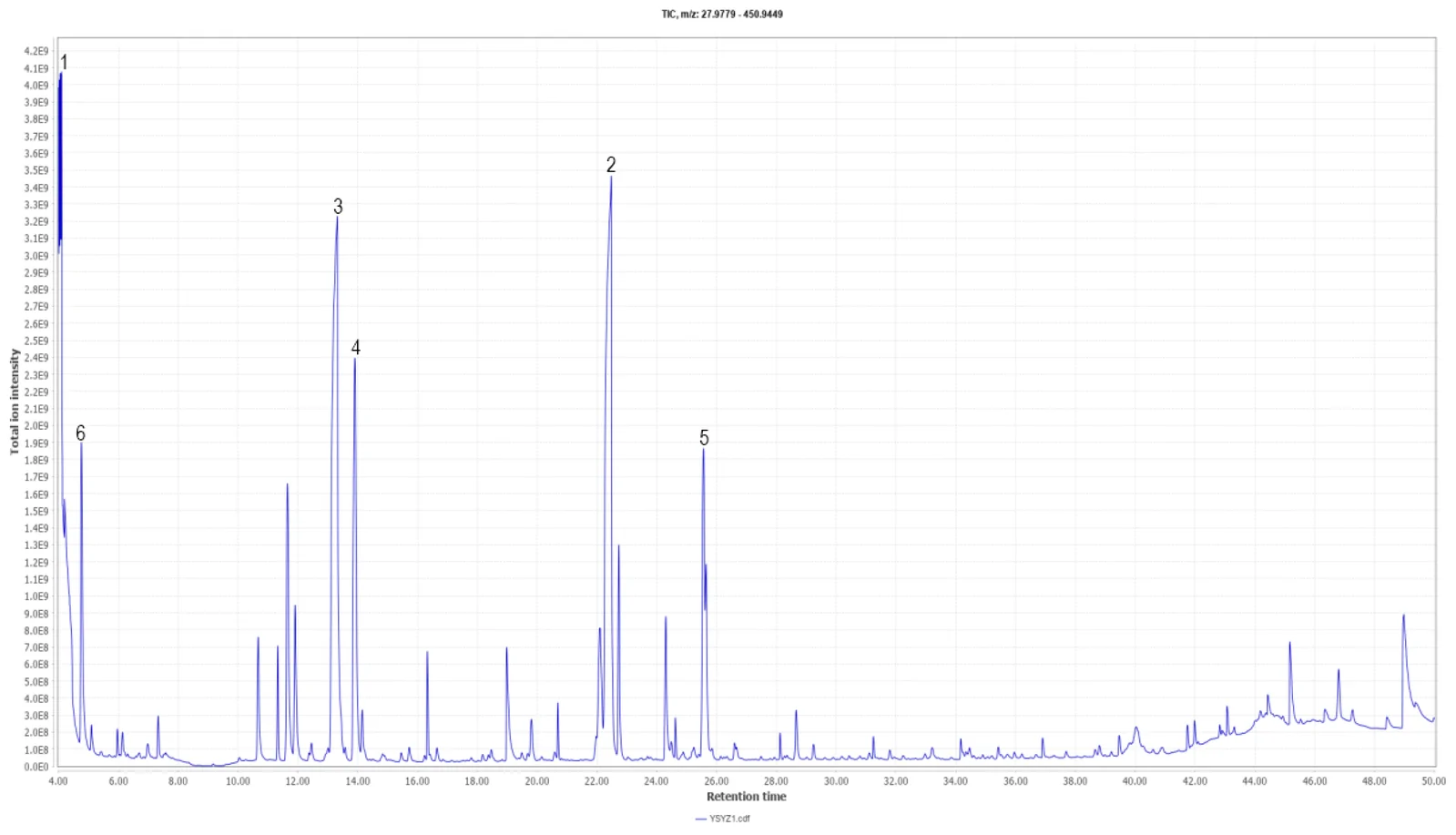
Total ion chromatogram (TIC) of the ethyl acetate ether-phase extract of *Spuriopinella brachycarpa* (Kom.) Kitag. seeds.

The total ion chromatograms (TICs) obtained from GC-MS analysis of each organic-phase extract are shown in Figure 1, Figure 2, Figure 3 and Figure 4. A total of 65 compounds were identified across all extracts (22, 14, 13, and 16 from the methanol, diethyl ether, petroleum ether, and ethyl acetate phases, respectively) based on the criteria described in Section 2.3. The relative signal intensity (RPA) and classification of these compounds are summarized in Table 2, Table 3, Table 4 and Table 5. The organic compounds detected in the extracts were classified according to the Human Metabolome Database (HMDB, version 5.0).

#### 2.2.1. Types of Organic Compounds Contained in Methanol-Phase Extract of *S. brachycarpa* Seeds

After GC-MS analysis, the total ion chromatogram (TIC) of the concentrated sample of the seed methanol-phase extract was compared with the standard chromatogram, and the relative signal intensity (RPA) was calculated according to the percentage of the peak area of the substance to the total peak area. Twenty-two organic compounds with great similarity were screened and are listed in Table 2. The organic compounds in the concentrated sample of the methanol-phase extract of *S. brachycarpa* seeds mainly include the following: pyrrolidone substances, the total relative signal intensity (RPA) of which is 7.39%; there are three kinds of pyrazines, and the total relative signal intensity (RPA) is 0.79%; one organic acid (acetic acid, 9.00%); three kinds of organic oxides, the total relative signal intensity (RPA) of which is 0.05%; two kinds of benzene compounds, the total relative signal intensity (RPA) of which is 4.75%; one lactone (0.63%), one phenol (1.29%), one monoterpene (1.02%), one fatty acid (1.50%) and one organic sulfide (0.01%); there are four substances with no specific classification information in the HMDB database, and their relative signal intensity (RPA) is 9.46% (Table 2) (Figure 1).

#### 2.2.2. Types of Organic Compounds Contained in Ether-Phase Extract of *S. brachycarpa* Seeds

After GC-MS analysis of concentrated samples of the ether-phase extract of *S. brachycarpa* seeds, the total ion chromatogram (TIC) of the ether-phase extract of seeds was compared with the standard chromatogram, and 14 organic compounds with great similarity were screened and are listed in Table 3. The main organic compounds are in the concentrated samples of the ether-phase extract of *S. brachycarpa* Eight monoterpenoids were detected in the diethyl ether phase, including limonene (3.08%), myrcene (1.05%), β-phellandrene (1.72%), camphene (0.52%), α-pinene (0.29%), sabinene (0.30%), benzene 1,3-bis(1,1-dimethylethyl)- (0.44%), and terpinolene (0.08%) (Table 3) (Figure 2).

#### 2.2.3. Types of Organic Compounds Contained in Petroleum Ether-Phase Extract of *S. brachycarpa* Seeds

After GC-MS analysis of concentrated samples of the petroleum ether-phase extract of *S. brachycarpa* seeds, the total ion chromatogram (TIC) of the petroleum ether-phase extract of seeds was compared with the standard chromatogram, and 13 organic compounds with great similarity were screened and are listed in Table 4. The organic compounds in the concentrated samples of the petroleum ether-phase extract of *S. brachycarpa* seeds showed seven monoterpenoids (including myrcene 3.19%, camphene 2.27%, α-pinene 1.76%, sabinene 1.36%, β-pinene 0.77%, benzene 1,3-bis(1,1-dimethylethyl)- 0.41%, and β-thujene 0.25%) and one hydrocarbon, γ-terpinene (13.90%) (Table 4) (Figure 3).

#### 2.2.4. Types of Organic Compounds Contained in Ethyl Acetate-Phase Extract of *S. brachycarpa* Seeds

After GC-MS analysis of concentrated samples of the seed ethyl acetate-phase extract, the total ion chromatogram (TIC) of the seed ethyl acetate-phase extract was compared with the standard chromatogram, and the relative signal intensity (RPA) was calculated according to the percentage of the peak area of the substance to the total peak area. Sixteen organic compounds with greater similarity were screened and are listed in Table 5. The main organic compounds in the concentrated samples from the ethyl acetate-phase extract of *S. brachycarpa* seeds are as follows: five monoterpenoids (3.59%); two kinds of sesami (3.70%); two kinds of fatty acids (3.37%); one organic acid (1.27%). There are also seven substances with no specific classification information in the HMDB database, and the total relative signal intensity (RPA) is 12.32% (Table 5) (Figure 4).

### 2.3. Screening of Metabolites of Organic Compounds Contained in Organic-Phase Extracts of S. brachycarpa Seeds

**Figure 5:**
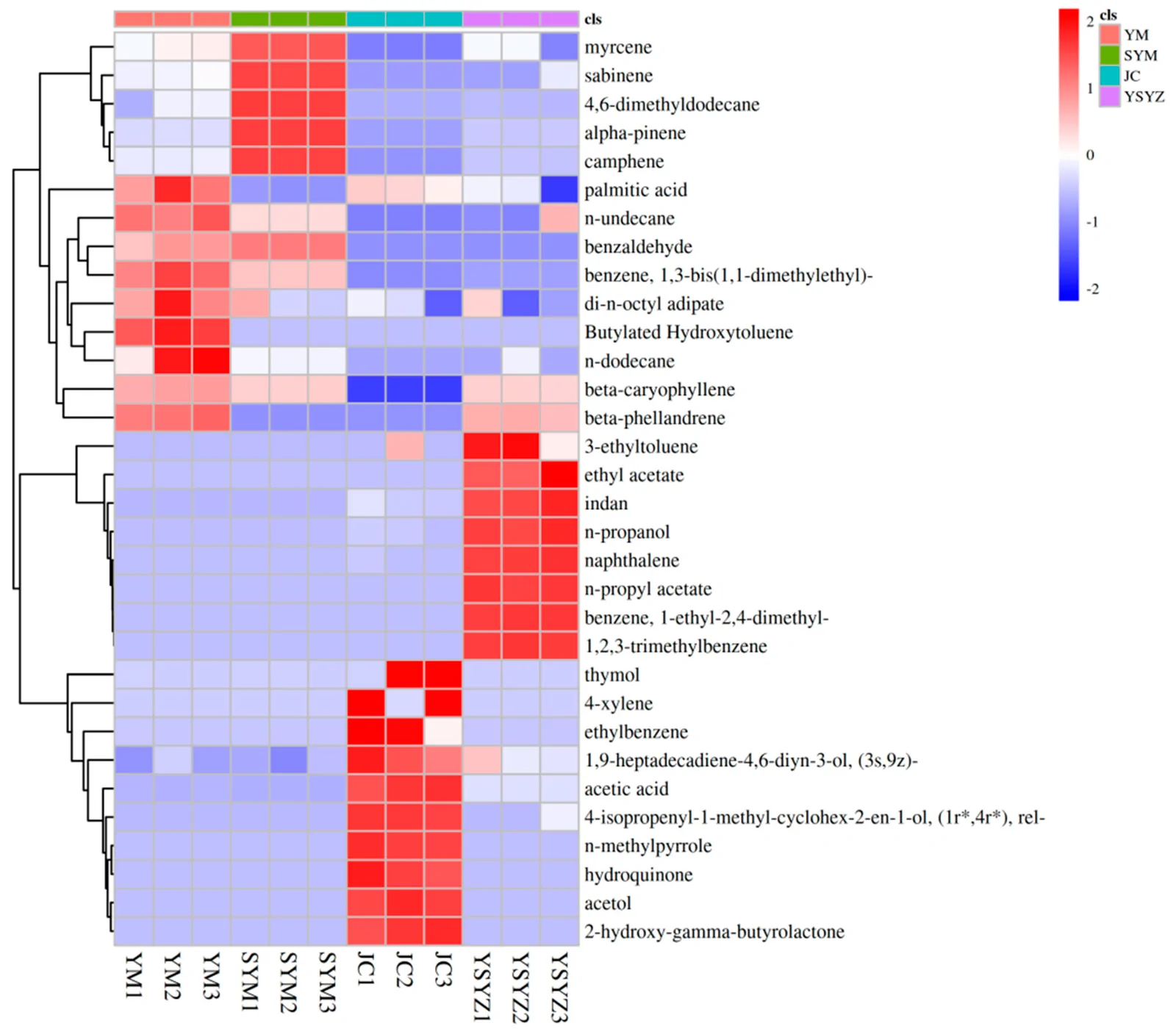
Heat map of substance distribution of extracts from each organic phase of extracts of *Spuriopinella brachycarpa* (Kom.) Kitag seeds.

In order to show more intuitively the content differences of organic compounds extracted from the organic-phase extracts of *S. brachycarpa* seeds and the content differences of various substances in the organic-phase samples, classification statistics and cluster analysis were carried out on the detected extract samples according to groups. Agglomerate hierarchical clustering is used, the dataset is scaled by pheatmap package in R (v3.3. 2), and the hierarchical cluster total ion chromatogram (TIC) of relative quantitative values of metabolites is obtained, as shown in Figure 5. The horizontal is sample grouping information, the vertical is metabolite information, and the color block legend is the expression amount obtained after standardization treatment. The redder the color, the higher the expression amount, and purple means a lower expression amount.

Thymol, 4-xylene, ethylbenzene, acetic acid, 1,9-heptadecadien-4,6-diyn-3-ol, acetic acid, 4-isopropenyl-1-1-methyl-cyclohexan-2-en-1-ol, n-methylpyrrole, hydroquinone, acetone alcohol, 2-hydroxy-γ-butyrolactone accumulated to high levels in the methanol-phase extract of *S. brachycarpa* seeds. The metabolites accumulated to high levels in the ether phase were palmitic acid, n-undecane, benzaldehyde, 1, -bis (1,1-dimethylethyl) benzene, di-n-octyl adipate, butadiene hydroxytoluene, n-dodecane, Sesquiterpene and β-hydroxycelene (Figure 5).

The organic compounds extracted from the organic-phase extracts of *S. brachycarpa* seeds were screened for differential metabolites, and the screened substances and their relative signal intensity (RPA) are presented in Table 6 below. It can be seen from the table that the metabolites with higher relative signal intensity (RPA) in the methanol phase mainly include the following 12 substances: N-methylpyrrole (6.34%), acetone alcohol (4.51%), 2-hydroxy-γ-butyrolactone (2.79%), hydroquinone (1.29%), 4-isopropenyl-1-methyl-cyclohexan-2-en-1-ol (1.02%), 1,9-heptadecadien-4,6-diyn-3-ol (1.31%), palmitic acid (1.50%), n-dodecan (0.14%), ethylbenzene (1.83%), 4-xylene (3.56%), thymol (1.89%) and di-n-octyl adipate (0.09%).

The main metabolites with higher relative signal intensity (RPA) in the ether phase were camphene (0.47%), Sesquiterpene (1.55%), β-hydroxycelene (1.72%), benzaldehyde (0.32%), benzene, 1,3-bis (1,1-dimethylethyl) (0.44%), 4,6-Alkane (0.07%), (0.30%), laurene (1.05%), 1,9-heptadecadien-4,6-diyn-3-ol (0.40%), n-undecane (0.10%), butadiene (1.29%), di-octyl adipate (0.23%), palmitic acid (1.49%), 4-xylene (0.01). The total relative signal intensity (RPA) of these substances in the ether phase is 9.45%. Among them, N-methylpyrrole (0.08%), acetone alcohol (4.51%), 2-hydroxy-γ-butyrolactone (2.79%), hydroquinone (1.29%) and ethylbenzene (1.83%) were expressed only in the methanol phase.

### 2.4. RNA Sequencing Quality

**Figure 6:**
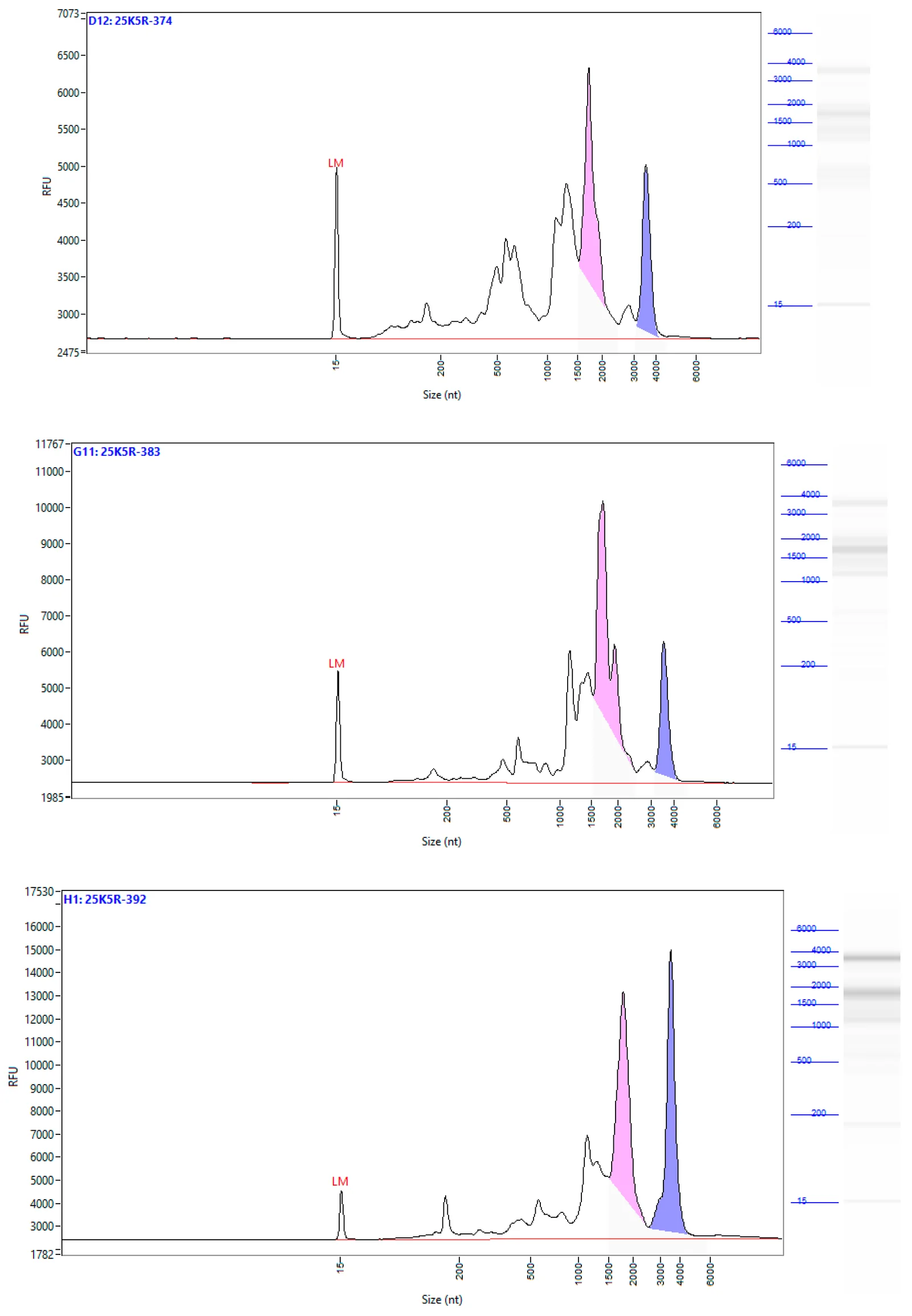
RNA integrity assessment of *Spuriopinella brachycarpa* (Kom.) Kitag. seeds at different stages. Note: Assessed by Agilent Bioanalyzer 2100 capillary electrophoresis. D1, M1, and R1 represent one biological replicate each from the dormancy (Dormancy), mid-germination (Mid), and germination-release (Release) stages, respectively; the other two biological replicates showed consistent quality control results. All samples exhibited clear 28S and 18S ribosomal RNA peaks (located at approximately 3000 nt and 2000 nt, respectively), with RIN values of 5.20, 7.00, and 7.10, respectively, and no obvious degradation smearing, indicating good RNA integrity that met the standards for transcriptome sequencing library construction.

Total RNA extracted from each sample was assessed via Agilent Bioanalyzer 2100 capillary electrophoresis. All samples exhibited distinct 28S and 18S ribosomal RNA peaks without obvious degradation smearing, with RIN values ranging from 5.00 to 8.20 (Figure 6), indicating good RNA integrity that met the standards for transcriptome sequencing library construction. In this study, the Illumina NovaSeq 6000 platform was employed for high-throughput sequencing.

Nine cDNA libraries (Dormancy1-3, Mid1-3, Release1-3) were subjected to paired-end sequencing to resolve the expression profile dynamics during the seed dormancy maintenance → mid-germination → germination-release process. After filtering the raw sequencing data with fqtrim, a total of 3.77 × 10^8^ valid reads were obtained, amounting to 55.89 Gb. The valid reads per sample ranged from 3.51 × 10^7^ to 4.88 × 10^7^, with valid percentages of 96.11–98.42%, Q20 ≥ 99.20%, Q30 ≥ 96.22%, and GC content of 46.14–52.79% (Table 7). These metrics indicate sufficient sequencing depth and high base accuracy, meeting the requirements for subsequent de novo assembly.

After quality control, filtering, and de novo assembly of the sequencing data, a total of 109,718 unigenes and 307,034 transcripts were obtained. As shown in Table 8, the average GC content of unigenes was 49.86%, and that of transcripts was 48.96%, indicating a relatively balanced sequence composition consistent with typical plant transcriptome characteristics.

In terms of length distribution, unigenes ranged from 201 bp to 23,082 bp, with a median length of 479 bp and an N50 length of 1506 bp; transcripts ranged more broadly from 176 bp to 23,082 bp, with a median length of 869 bp and an N50 length of 1673 bp. Overall, transcripts exceeded unigenes in both length and quantity, suggesting that the assembly results were relatively complete and capable of adequately covering full-length transcript information. Additionally, the total assembled bases for unigenes and transcripts reached 95,556,372 bp and 353,290,335 bp, respectively, providing abundant sequence resources for subsequent functional annotation, differential expression analysis, and key gene screening.

**Figure 7:**
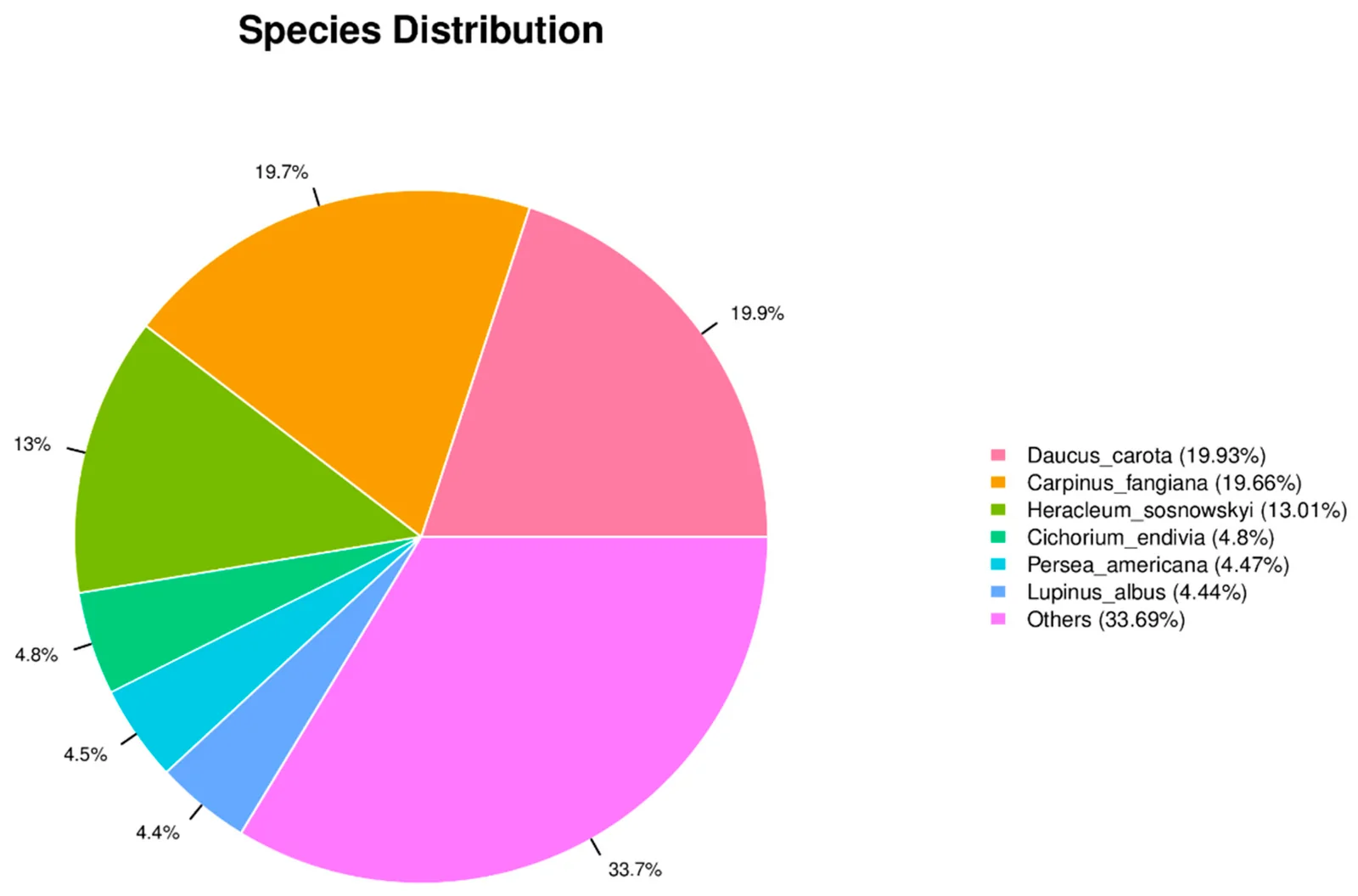
Pie chart of species annotation for *Spuriopinella brachycarpa* (Kom.) Kitag. seeds based on the NR database.

NR annotation results showed that a total of 65,392 unigenes obtained homologous matches (Figure 7). Among these, the species with the highest match rate was carrot (Daucus carota), with 13,035 annotated sequences accounting for 19.93% of the total annotations, followed by Carpinus fangiana (12,853 sequences, 19.66%), Heracleum sosnowskyi (8506 sequences, 13.01%), cultivated chicory (Cichorium endivia, 3142 sequences, 4.80%), avocado (Persea americana, 2923 sequences, 4.47%), and white lupin (Lupinus albus, 2901 sequences, 4.44%). The remaining species each had fewer than 2000 annotated sequences, collectively accounting for 33.69%. This species distribution suggests that the transcriptome of this study exhibits high sequence conservation with Apiales and Fabaceae plants, providing a reliable reference for subsequent functional gene validation and comparative species studies.

### 2.5. Functional Annotation of Unigenes

A total of 109,718 non-redundant unigenes were subjected to homologous alignment against seven major databases (DIAMOND, E-value ≤ 1 × 10^−5^). The annotation success rates were as follows(Table 9): NR 59.60%, eggNOG 78,481 sequences (71.53%), GO 62.52%, Pfam 61.74%, KEGG 21.52%, Swiss-Prot 50.98%; additionally, 1513 transcription factors (TFs, 1.38%) were predicted. The overall annotation coverage exceeded 70%, meeting the requirements for subsequent functional enrichment and pathway analysis of differentially expressed genes.

### 2.6. Differential Gene Expression Analysis

**Figure 8:**
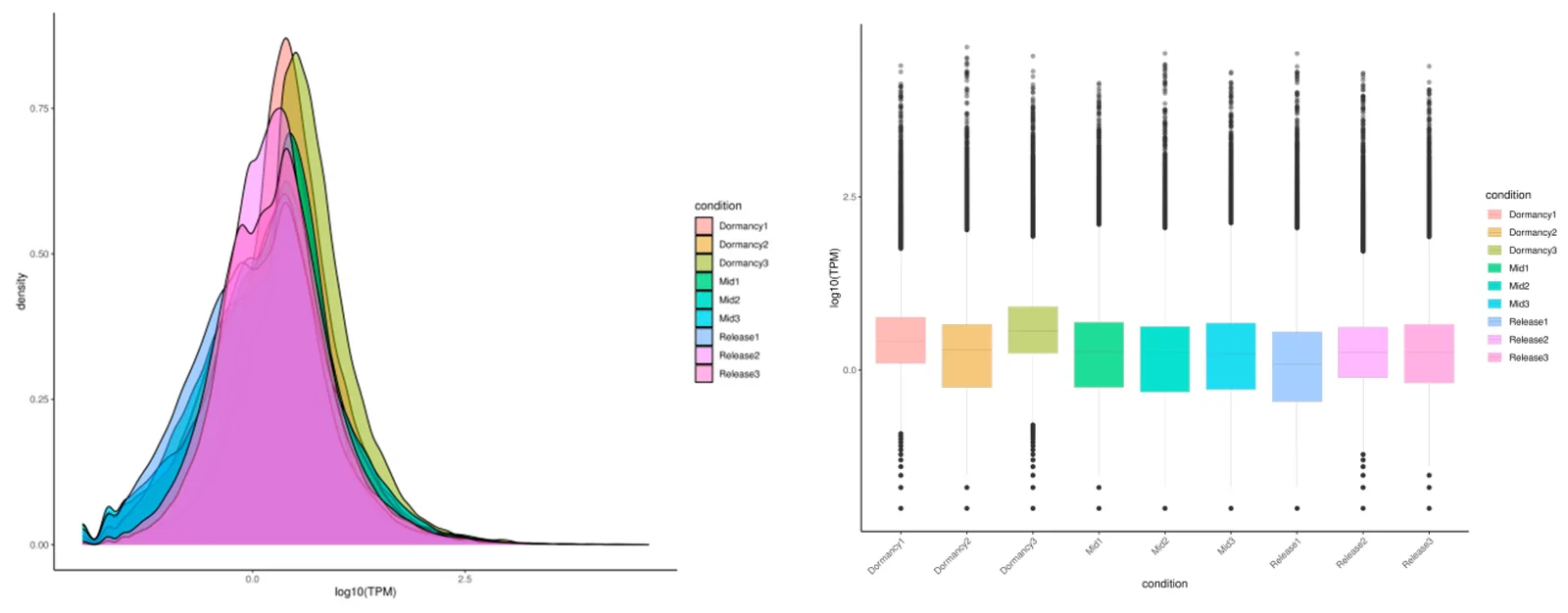
Differential gene expression analysis of *Spuriopinella brachycarpa* (Kom.) Kitag. seeds.

After TPM normalization of 109,718 non-redundant genes, expression distribution box plots and density curves were generated. Box plot analysis showed that the median expression level of the three Dormancy biological replicates (log_10_TPM ≈ 1.2) was slightly higher than that of the Mid and Release groups, indicating overall higher transcriptional activity during the dormancy stage. Density curves further revealed that all samples exhibited typical bimodal distributions, with the low-expression peak (log_10_TPM < 0.5) primarily composed of rare transcripts, and the high-expression peak (log_10_TPM 1.5–2.5) concentrated with housekeeping and functional regulatory genes. Neither plot showed obvious batch effects, confirming the reliability of sequencing and quantification results and providing a high-quality data foundation for subsequent differential expression analysis (Figure 8).

### 2.7. Differential Gene Screening

**Figure 9:**
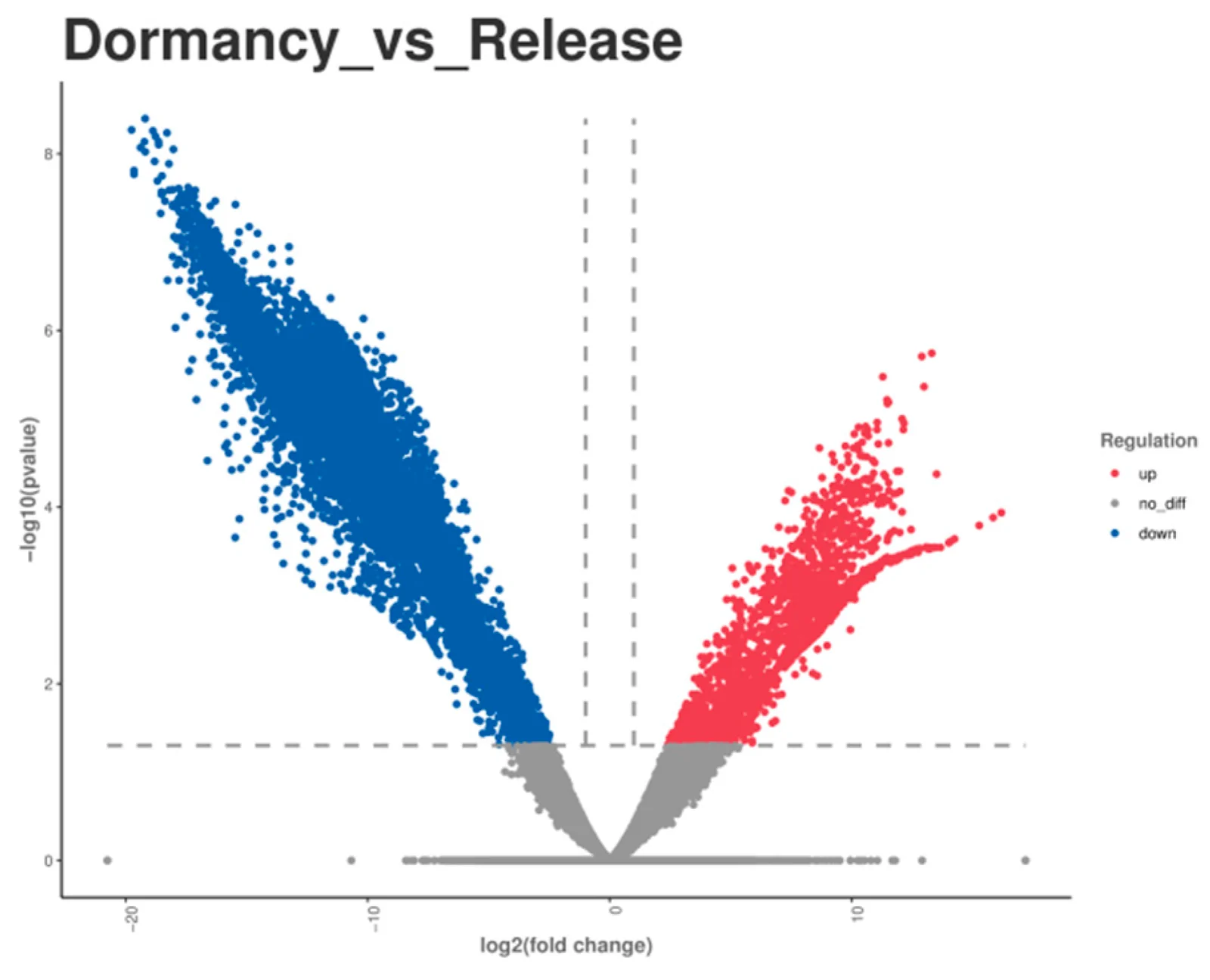
Volcano plot of differentially expressed unigenes in *Spuriopinella brachycarpa* (Kom.) Kitag. Seeds.

According to the volcano plot, a large number of differentially expressed genes (DEGs) were identified between the Dormancy and Release comparison groups. Significantly upregulated genes (red) were mainly distributed in the region where log_2_(fold change) > 1, with relatively large numbers, indicating that a substantial number of genes were activated during the transition from dormancy to release. Significantly downregulated genes (blue) were mainly distributed where log_2_(fold change) < −1, with even greater numbers than upregulated genes, and some genes showed extremely significant downregulation (log_2_FC reaching −20), suggesting that these genes were highly expressed during dormancy but strongly suppressed after release. Furthermore, a large number of DEGs reached extremely high statistical significance levels (−log_10_(*p*-value) > 8), indicating highly significant gene expression differences between the two groups. Non-significantly differential genes (gray) were concentrated in the central region with minimal expression changes. Overall, these results reveal dramatic and extensive transcriptomic reprogramming between dormancy and release states, providing an important candidate gene set for in-depth analysis of the molecular mechanisms underlying this biological process (Figure 9).

### 2.8. ggNOG Functional Annotation of Unigenes

**Figure 10:**
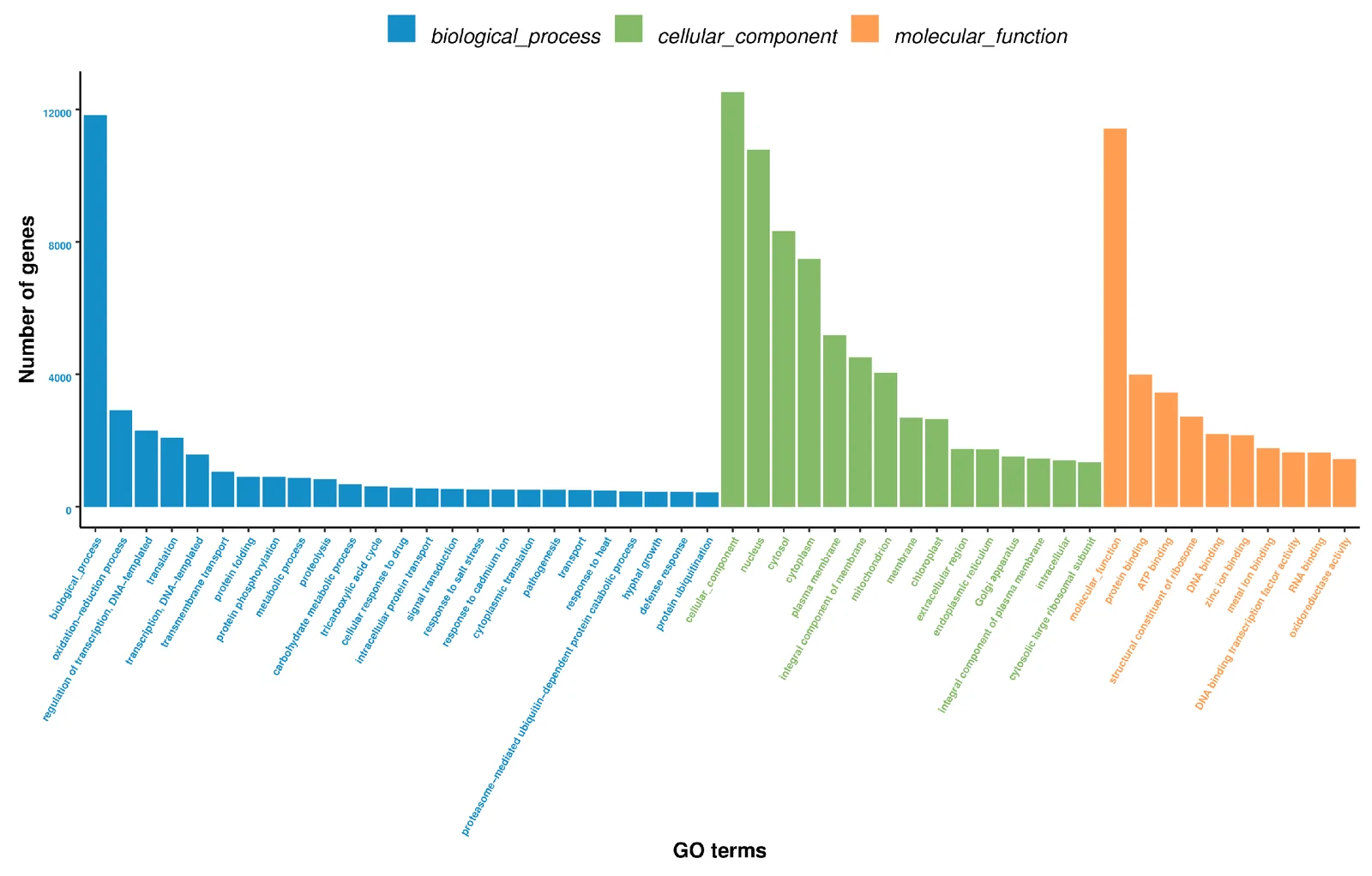
Bar chart of gene (GO) annotation for *Spuriopinella brachyara* (Kom.) Kitag. seeds.

**Figure 11:**
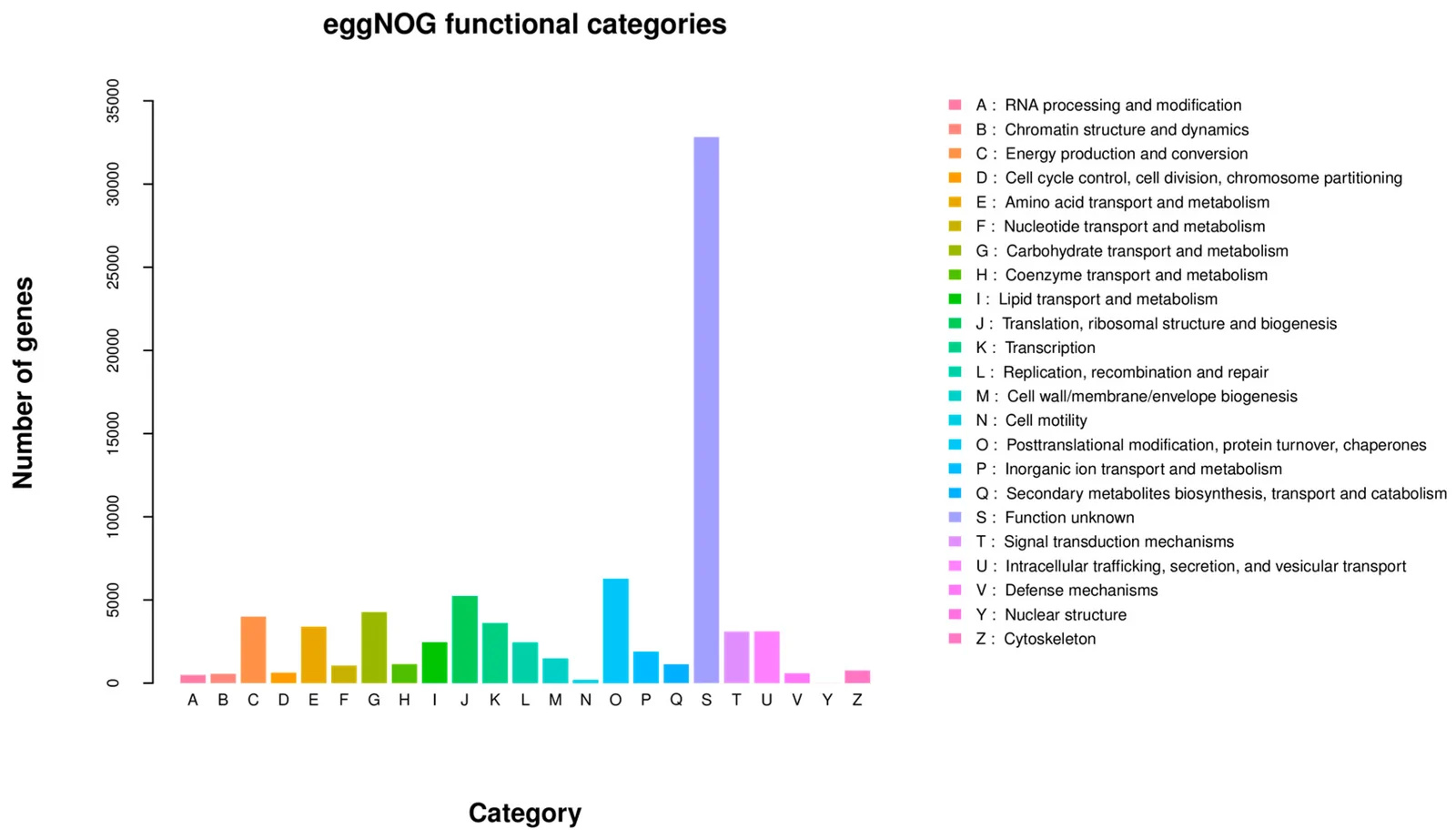
Bar chart of eggNOG functional classification for *Spuriopinella brachyara* (Kom.) Kitag. Seeds.

eggNOG functional annotation results showed that the 78,481 annotated unigenes were classified into 23 COG functional categories, covering multiple biological modules including cellular metabolism, information storage and processing, and cellular processes and signaling (Figure 10). Among these, function unknown was the most abundant category, with 32,877 unigenes accounting for 41.89% of the total annotations, followed by posttranslational modification, protein turnover, chaperones with 6288 sequences (8.01%); translation, ribosomal structure and biogenesis with 5274 sequences (6.72%); carbohydrate transport and metabolism with 4345 sequences (5.54%); and energy production and conversion with 4255 sequences (5.42%). Additionally, transcription and signal transduction mechanisms were annotated with 3617 (4.61%) and 3165 (4.03%) sequences, respectively. This distribution pattern indicates that although a large number of gene functions remain to be elucidated, energy metabolism, protein synthesis, and primary metabolic activities are highly active in the samples, providing an important foundation for subsequent key functional gene mining and metabolic pathway construction (Figure 10 and Figure 11).

### 2.9. GO Functional Annotation of Unigenes

GO functional annotation results showed that 68,592 unigenes were annotated to 50 GO terms, covering three major categories: biological_process, cellular_component, and molecular_function. In the molecular_function category, protein binding was the most abundant term with 3986 annotated unigenes, followed by ATP binding (3450 unigenes) and structural constituent of ribosome (2723 unigenes). In the cellular_component category, nucleus had the highest number of annotated genes, followed by cytosol (8328 unigenes) and cytoplasm (7493 unigenes). In the biological_process category, the oxidation reduction process was the most abundant with 2911 annotated genes, followed by regulation of transcription, DNA-templated (2295 unigenes) and translation (2084 unigenes). This distribution pattern indicates active energy metabolism, abundant membrane system structures, and frequent gene expression regulation processes in the current samples, providing clear directions for subsequent screening of key functional genes.

### 2.10. Unigene KEGG Functional Annotation

**Figure 12:**
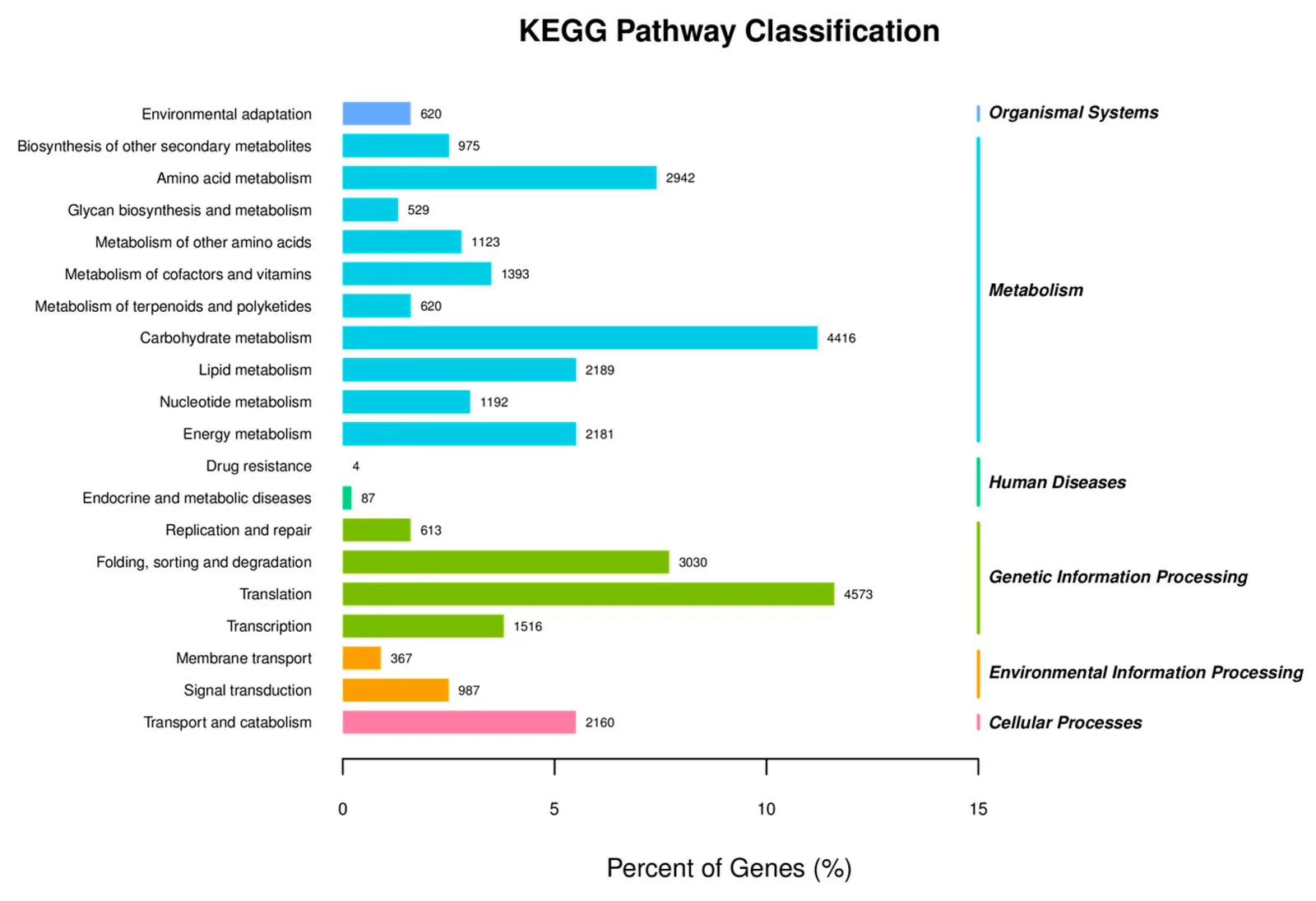
Bar chart of gene KEGG annotation for *Spuriopinella brachyara* (Kom.) Kitag. Seeds.

KEGG pathway classification revealed that among 54,862 unigenes, 39,499 were successfully annotated to six major top-level branches (Figure 12). Metabolism accounted for the largest proportion (23,831 unigenes, representing 60.33% of the total annotated sequences). Within this branch, the most enriched secondary pathways were carbohydrate metabolism (4416 unigenes), amino acid metabolism (2942 unigenes), and lipid metabolism (2189 unigenes). Within the genetic information processing branch, translation (4573 unigenes) and folding, sorting and degradation (3030 unigenes) were the predominant categories. The cellular processes branch was mainly concentrated in transport and catabolism (2160 unigenes).

### 2.11. Transcription Factor Annotation

**Figure 13:**
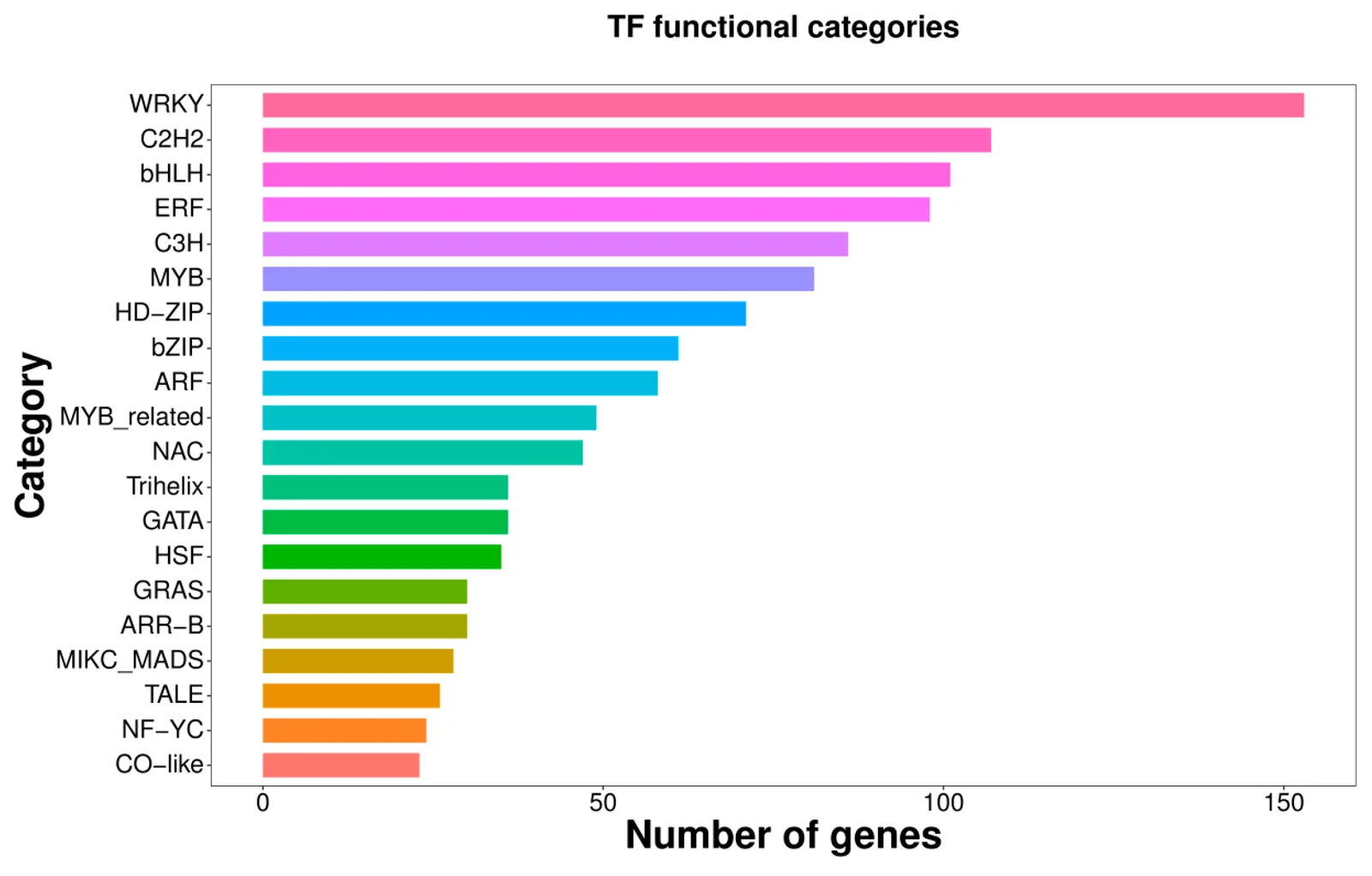
Statistical chart of transcription factors in *Spuriopinella brachyara* (Kom.) Kitag. Seeds.

Transcription factor (TF) family annotation was performed on the transcriptome data (Figure 13), and a total of 1513 transcription factor genes were identified, distributed across 54 distinct TF families. In terms of family distribution, the WRKY family was the most abundant, comprising 153 genes and accounting for 10.1% of the total transcription factor genes, followed by the C2H2 family (107 genes, 7.1%), the bHLH family (101 genes, 6.7%), the ERF family (98 genes, 6.5%), and the C3H family (86 genes, 5.7%). These five dominant families collectively contained 545 genes, representing 36.0% of the total, indicating a marked imbalance in family distribution. The top 10 families encompassed 865 genes, reaching 57.2% of the total, while the remaining 44 families accounted for only 42.8%. Further analysis revealed that only three super-families contained more than 100 genes (WRKY, C2H2, and bHLH), six large families ranged from 50 to 99 genes, seven medium-sized families ranged from 30 to 49 genes, nineteen small families ranged from 10 to 29 genes, and nineteen rare families contained fewer than 10 genes, exhibiting a typical long-tail distribution pattern.

### 2.12. Differential Unigene GO Enrichment

**Figure 14:**
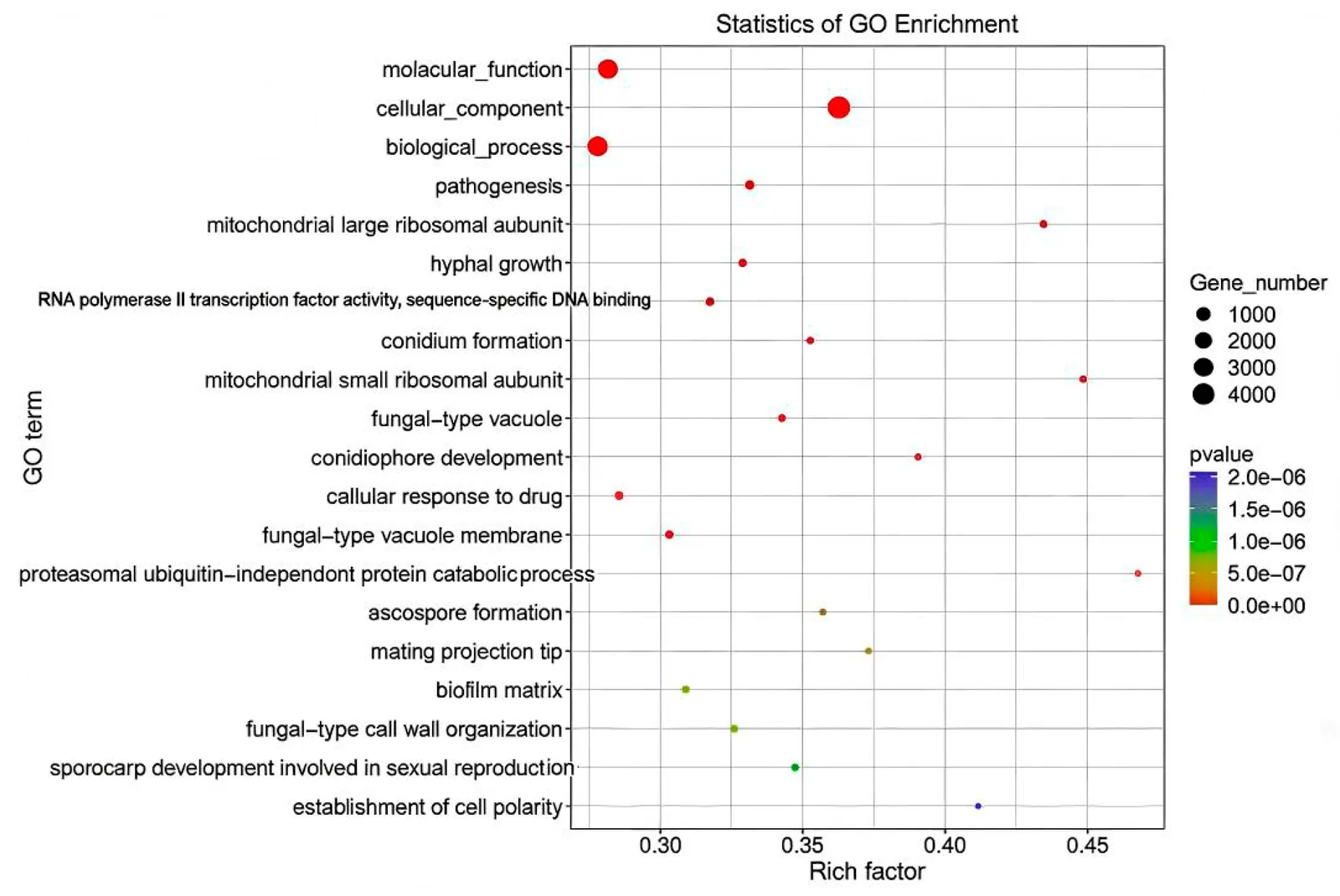
Statistical chart of GO enrichment in *Spuriopinella brachyara* (Kom.) Kitag. seeds. Note: GO terms of microbial origin (e.g., hyphal growth, ascospore formation, fungal-type vacuole) were returned by the annotation pipeline.

GO functional enrichment analysis was performed on differentially expressed genes between the Dormancy and Release stages (Figure 14). In the molecular function category, RNA polymerase II transcription factor activity and sequence-specific DNA binding (89 genes, rich factor 0.33) and pathogenesis-related functions (76 genes, rich factor 0.34) were significantly enriched. In the cellular component category, the mitochondrial large ribosomal subunit (76 genes, rich factor 0.44) and the mitochondrial small ribosomal subunit (67 genes, rich factor 0.45) were significantly enriched, exhibiting the highest rich factor values among all entries, suggesting that mitochondrial function underwent substantial changes during the dormancy-to-release transition. In the biological process category, the proteasome ubiquitin-independent protein catabolic process contained approximately 45 annotated genes and showed the highest rich factor (0.46), indicating that protein degradation metabolism was strongly activated during the Release stage.

Some GO terms returned by the annotation pipeline were of microbial origin and were excluded from interpretation due to their biological implausibility in plant seed transcriptomes. This limitation likely reflects non-plant sequences in the NR database used for annotation.

### 2.13. Differential Unigene KEGG Enrichment Scatter Plot

**Figure 15:**
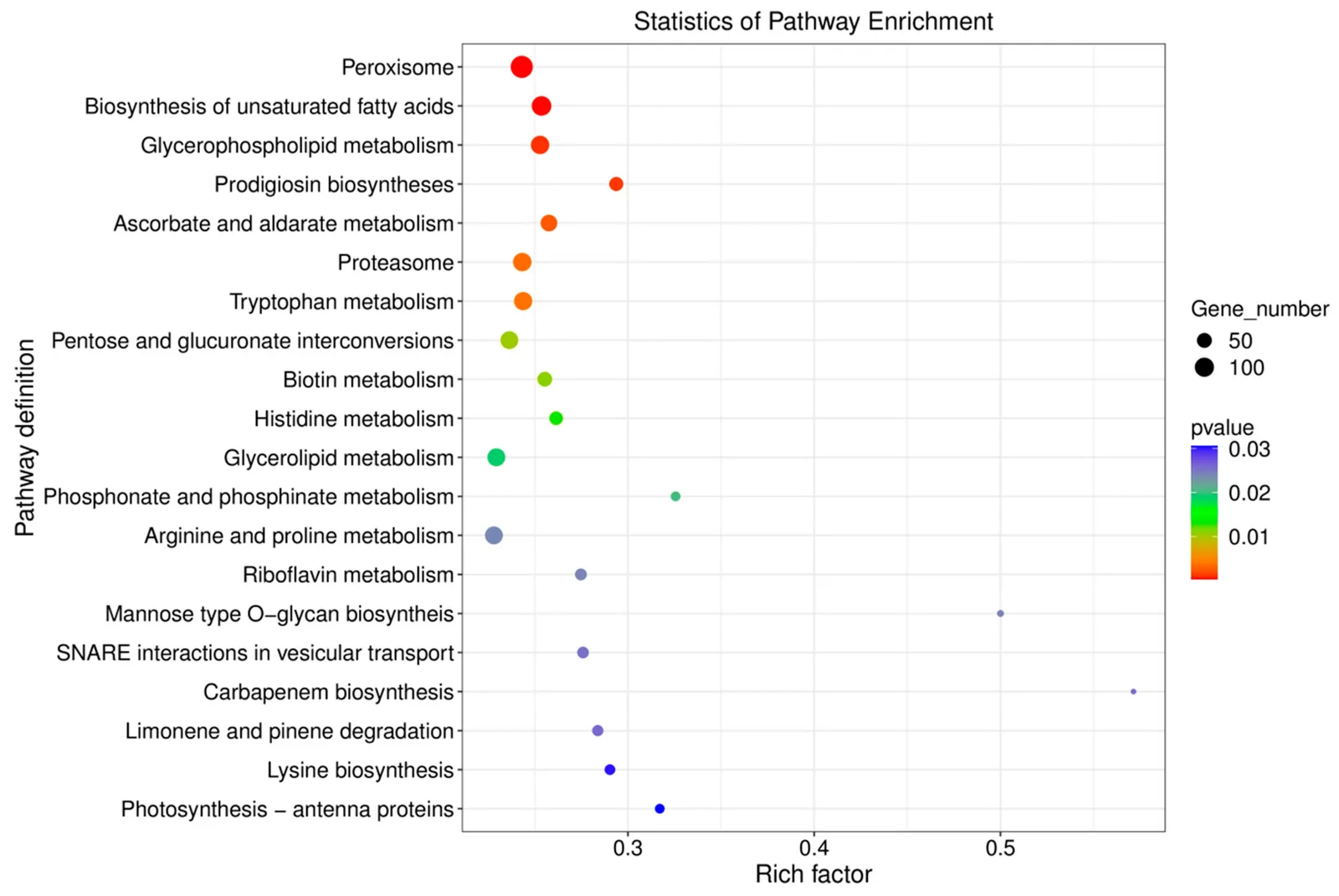
Scatter plot of Unigene KEGG enrichment for differences in arugula seed composition in *Spuriopinella brachyara* (Kom.) Kitag. seeds.

To investigate metabolic differences between the Dormancy and Release stages, KEGG pathway enrichment analysis was performed on differentially expressed genes (Figure 15). The results showed that a total of 20 metabolic pathways were significantly enriched (*p* < 0.05), among which peroxisome metabolism, unsaturated fatty acid biosynthesis, and glycerophospholipid metabolism exhibited the most pronounced differences.

The peroxisome pathway (Peroxisome, map04146) was the most significantly enriched pathway (*p* = 2.36 × 10^−4^), with a rich factor of approximately 0.22, involving 103 differentially expressed genes. The vast majority of genes in this pathway were significantly upregulated during the Release stage, including key enzymes of fatty acid β-oxidation such as ACX1 (acyl-CoA oxidase), POT1 (3-ketoacyl-CoA thiolase), and ECH1 (enoyl-CoA hydratase), as well as antioxidant defense-related genes such as CAT1 (catalase), SOD1/2 (superoxide dismutase), and PEX family genes (peroxisome biogenesis-related).

The biosynthesis of the unsaturated fatty acids pathway (Biosynthesis of unsaturated fatty acids, map01040) was also highly significant (*p* = 3.32 × 10^−4,^ rich factor ≈ 0.24), containing 87 differentially expressed genes. This pathway was enriched with multiple fatty acid desaturase genes, including FAD2, FADX, and FAH12, which are responsible for catalyzing the conversion of oleic acid to linoleic acid and polyunsaturated fatty acids. Notably, this pathway exhibited significant gene overlap with the peroxisome pathway, with genes such as POT1 and ACX1 participating simultaneously in both fatty acid oxidation and unsaturated fatty acid biosynthesis, suggesting that membrane lipid remodeling during the Release stage is closely associated with energy metabolism.

The glycerophospholipid metabolism pathway (Glycerophospholipid metabolism, map00564) ranked third (*p* = 9.87 × 10^−4^, rich factor ≈ 0.25), involving 76 differentially expressed genes, mainly including phospholipid synthases (such as PSD1 and PGS1), phospholipases (such as PLA2 and LPP1), and choline/ethanolamine phosphotransferase (CEPT). The significant enrichment of this pathway indicates that membrane system reconstruction and repair activities are enhanced during the release stage.

Additionally, pathways, including prodigiosin biosynthesis (Prodigiosin biosyntheses, map00333), ascorbate and aldarate metabolism (Ascorbate and aldarate metabolism, map00053), proteasome (Proteasome, map03050), and tryptophan metabolism (Tryptophan metabolism, map00380), also exhibited significant differences (*p* < 0.01), involving biological processes such as secondary metabolism, antioxidant defense, protein turnover, and amino acid metabolism, respectively.

### 2.14. Differential Gene Statistics

**Figure 16:**
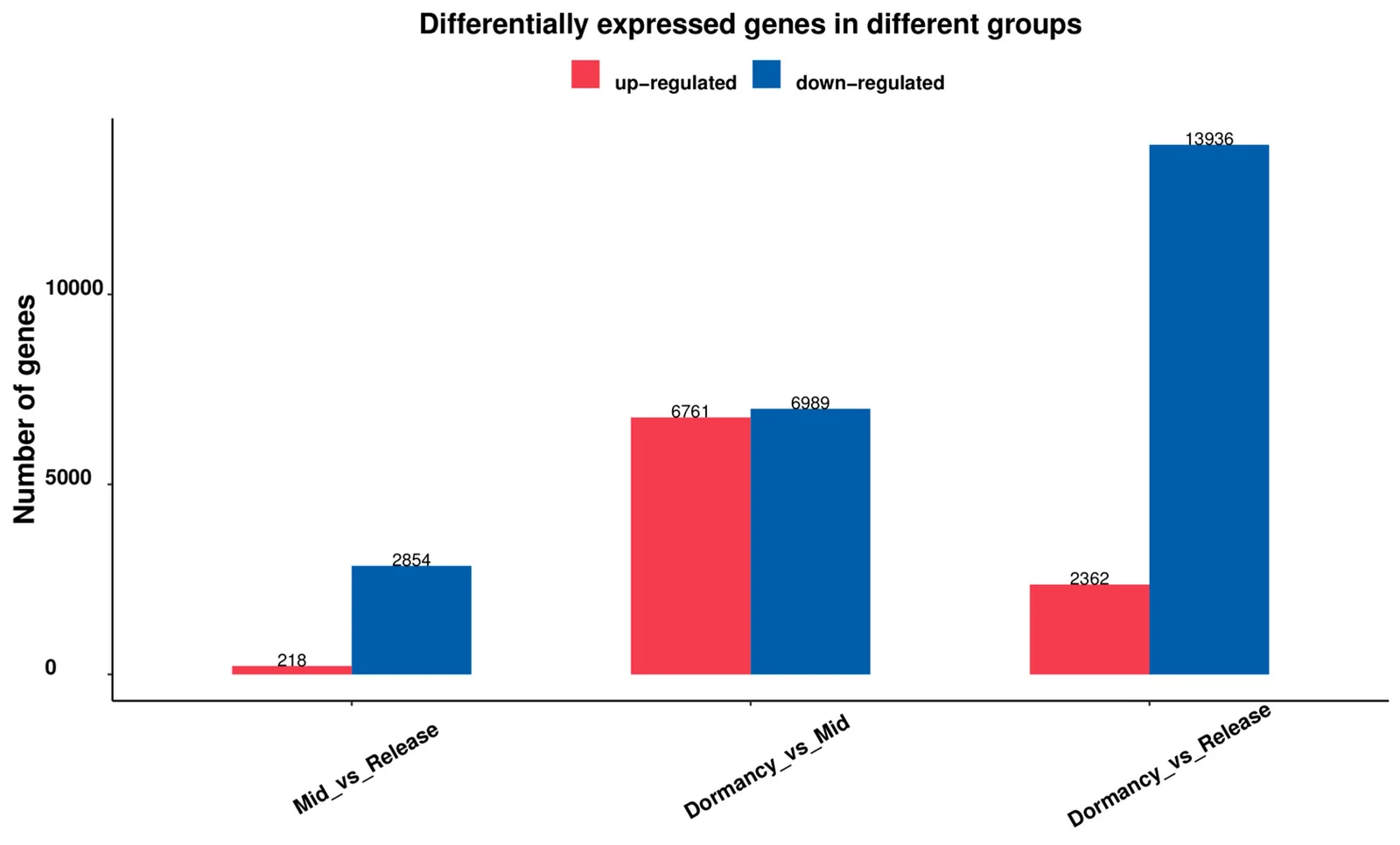
Bar chart of up/downregulated unigenes in *Spuriopinella brachyara* (Kom.) Kitag. Seeds.

Differential expression analysis (DESeq2, padj < 0.05 and |log_2_FC| ≥ 1) revealed that 13,750, 16,298, and 3072 differentially expressed genes (DEGs) were identified in the Dormancy-vs-Mid, Dormancy-vs-Release, and Mid-vs-Release comparisons, respectively (Figure 16). The bar chart statistics indicated that the Dormancy-to-Release comparison yielded the largest number of DEGs, suggesting a global transcriptomic shift from activation to suppression, or that a substantial proportion of genes were highly expressed during dormancy and subsequently downregulated upon germination. The intersection of the three comparison groups yielded 4628 core DEGs, among which 385 were continuously upregulated and 153 were continuously downregulated, and these genes prominently exceeded the thresholds of |log_2_FC| > 4 and padj < 10^−20^ in the extreme regions of the volcano plots (Figure 16), indicating that these genes exhibited conserved directional changes with high confidence and could serve as priority candidates for dissecting the key regulatory network underlying dormancy release.

### 2.15. Screening of DEGs Related to Seed Dormancy and Germination in S. brachycarpa 

During seed germination, differentially expressed genes within the plant hormone signal transduction pathway (map04075) exhibited significant temporal expression characteristics, involving ABA signaling, dormancy-stage highly expressed genes, and germination-stage downregulated genes (Table 10).

ABA Signal Regulation: The ABA receptor component PYL4-like (TRINITY_DN21355_c0_g1) was significantly downregulated at the mid-germination stage (log_2_FC = −3.22, *p* < 0.05), indicating that ABA perception activity diminished as germination progressed, which is consistent with the physiological transition of seeds from dormancy to germination. The attenuation of ABA signaling is a characteristic feature of seed germination, and the downregulation of ABA receptors identified in this study supports this trend.

Dormancy-Stage Highly Expressed Genes: Four genes were significantly upregulated during the dormancy stage, with notable variation in expression magnitude. The functionally uncharacterized gene TRINITY_DN1185_c0_g1 exhibited the greatest upregulation (log_2_FC = +7.71 in the DvsM group, *p* < 0.05), representing the highest expression level among all hormone signaling-related genes identified. The TRANSPORT RESPONSE 1-like protein-encoding gene (TRINITY_DN68528_c1_g1) was significantly upregulated in both the DvsM and DvsR comparisons (log_2_FC = +6.66 and +6.27, respectively, *p* < 0.05); its functional classification remains unclear, though its differential expression detected across both comparisons indicates a distinct temporal expression pattern. TRINITY_DN21468_c0_g1 (predicted protein) was concurrently upregulated (log_2_FC = +5.39, *p* < 0.05), sharing the dormancy-stage high-expression characteristic with the aforementioned genes. The scarecrow-like protein 28 (TRINITY_DN41542_c0_g2) showed a relatively lower upregulation magnitude (log_2_FC = +2.96, *p* < 0.05); this protein is annotated as PREDICTED in the database, and its specific function and relationship with GA signaling require further verification.

Germination-Stage Downregulated Genes: One functionally uncharacterized gene exhibited extremely significant suppressed expression at the mid-germination stage. TRINITY_DN12308_c0_g1 (log_2_FC = −10.82, *p* < 0.05) showed the greatest downregulation magnitude, representing the most significantly differentially expressed gene among all hormone signaling-related genes. The downregulation exceeded 10-fold, forming a striking contrast with the dormancy-stage highly expressed genes (upregulated by 5–7-fold), demonstrating a pronounced temporal expression shift.

## 3. Discussion

The metabolomic and transcriptomic datasets were analyzed in a complementary manner rather than through a fully integrated multi-omics framework; therefore, the present findings should be interpreted as correlative rather than causal.

### 3.1. Endogenous Inhibitors and the Physiological Component of MPD

Seeds of the Apiaceae family typically exhibit morphophysiological dormancy (MPD), which involves both underdeveloped embryos at dispersal and a physiological dormancy component. The physiological component of MPD is regulated by internal hormonal balance (notably ABA dominance) and requires specific environmental signals for release, such as warm followed by cold stratification or GA_3_ application [10,11]. While endogenous chemical inhibitors may strengthen the physiological component of MPD, they cannot be considered the primary cause of the morphological component (i.e., embryo underdevelopment) [12,13]. In this study, we identified endogenous inhibitors in *S. brachycarpa* seeds that showed inhibitory effects on Chinese cabbage seed germination and growth. However, it is important to note that these compounds likely contribute to, but are not the sole cause of, the physiological component of MPD. The morphological component of MPD—underdeveloped embryos requiring growth and differentiation—cannot be explained by chemical inhibition alone.

It is crucial to acknowledge that demonstrating inhibitory effects on Chinese cabbage seeds does not prove that these compounds are the primary cause of dormancy in *S. brachycarpa*. Chinese cabbage and *S. brachycarpa* represent different physiological backgrounds and may respond differently to the same compounds. The identified compounds could function as allelopathic substances that inhibit germination of competitor plants, or they may strengthen the physiological component of MPD in *S. brachycarpa* without being its primary cause [14]. Therefore, the present study provides evidence that these compounds can act as germination inhibitors but does not demonstrate that they are the key factors responsible for seed dormancy in *S. brachycarpa.*

In this experiment, the determination of germination inhibitors of *S. brachycarpa* seeds showed that there were endogenous inhibitors causing seed dormancy in *S. brachycarpa* seeds, mainly distributed in the methanol and ether phases. The inhibitory effect of the extracts of each organic phase on the germination and growth of Chinese cabbage seeds was as follows: methanol phase > ether phase > ethyl acetate phase > petroleum ether phase. The methanol-phase extracts of *S. brachycarpa* seeds show significant inhibition on the germination percentage, root length and seedling height of Chinese cabbage seeds, which is similar to the conclusion obtained in previous studies on Prunus mume [15], Platycodon grandiflorum [16], hawthorn [17] and sand mustard [18].

A large number of previous studies have shown that the common germination inhibitors affecting seed germination mainly include organic acids, phenols, aldehydes, unsaturated lactones, alkaloid and so on [19,20]. Han Baorui et al. (1999) extracted and isolated six germination inhibitors from American ginseng pulp, which were acetic acid, heptanoic acid, nonanoic acid, butyric acid, nonanal and isobutyric acid [21]. Shang Xulan found that there are germination inhibitors such as heptanic acid, caprylic acid, palmitic acid and diethyl phthalate in *Cyclocarya paliurus* seeds [22]. Wang Na and others found that palmitic acid and cinnamic acid inhibited the growth and germination of pea seeds [23]. Li Biane and others found that p-hydroxybenzaldehyde had inhibitory activity on the growth of wheat, barnyard grass, amaranth retroflexion and lettuce seedlings [24]. Wang Lianqun and others found that thymol can effectively inhibit the mycelial growth of *Fusarium graminearum* [25]. Shang Yan et al. found that HTPB had an obvious inhibitory effect on the root length and seedling height of lettuce, Chinese cabbage and radish [26].

Among the organic compounds identified in the four organic-phase extracts of *S. brachycarpa* seeds, the major constituents of the methanol phase (the most active fraction) were as follows: acetic acid (9.00%), N-methylpyrrole (6.34%), acetol (4.51%), 4-xylene (3.56%), 2-hydroxy-γ-butyrolactone (2.79%), palmitic acid (1.50%), 1,9-heptadecadiene-4,6-diyn-3-ol (1.31%), hydroquinone (1.29%), ethylbenzene (1.83%), thymol (1.89%), and 4-isopropenyl-1-methylcyclohex-2-en-1-ol (1.02%). Acetic acid was additionally detected in the ethyl acetate phase (1.27%), palmitic acid in the diethyl ether phase (1.49%), and benzaldehyde in the diethyl ether (0.32%) and petroleum ether (0.55%) phases. Among these, acetic acid, palmitic acid, benzaldehyde, and thymol have been reported to possess germination inhibitory activity in previous studies and represent priority candidates for bioassay-guided purification. Butylated hydroxytoluene (BHT), although detected at 1.29% in the diethyl ether phase, is a synthetic antioxidant commonly added to solvents and was, therefore, excluded from the candidate inhibitors; the aromatic hydrocarbons ethylbenzene and 4-xylene were likewise considered likely environmental contaminants rather than endogenous metabolites (see Section 4.3).

To sum up, this study presents the first systematic metabolic profiling of endogenous extracts from *S. brachycarpa* seeds, identifying compounds associated with germination inhibitory activity in heterologous bioassays. The detection of acetic acid, palmitic acid, benzaldehyde, and thymol as dominant constituents of the inhibitory methanol phase represents a novel metabolic finding for this species, providing a biochemical basis for understanding the physiological component of morphophysiological dormancy (MPD). While these compounds have been reported in other plant species, their implication in Apiaceae seed dormancy regulation has not been previously described. These compounds may strengthen the physiological component of MPD, but they cannot be considered the primary cause of MPD, which involves distinct morphological (embryo underdevelopment) and physiological components. The main types of identified compounds include phenols, ketones, esters, organic acids, and lignin. Among these, acetic acid, palmitic acid, benzaldehyde, and thymol are candidate compounds that may contribute to the inhibitory effect, though the specific endogenous inhibitory compounds responsible for seed dormancy in *S. brachycarpa* remain to be fully characterized. Further bioassay-guided fractionation and purification are needed to isolate the active components from the methanol- and ether-phase extracts, followed by direct testing of these purified compounds on *S. brachycarpa* embryos to confirm their role in inducing and maintaining seed dormancy. Due to the absence of bio-guided fractionation, equipment blank controls, and direct evidence linking these compounds to the physiological dormancy component of *S. brachycarpa*, this study does not claim that any single compound is solely responsible for the observed dormancy. According to the GC-MS-based metabolic profiling, the main types of endogenous inhibitors include phenols, ketones, esters, organic acids, and lignin. In this experiment, the methanol-phase extract had the strongest inhibitory effect, and the substances contained in the methanol-phase extract may be extract-associated compounds that showed inhibitory activity in a bioassay, whose causal roles remain to be verified, followed by the substances contained in the ether-phase extract. Among these substances, which kind of substances play a major role in the physiological dormancy of *S. brachycarpa* seeds and the relationship between them need further analysis and research.

The detection of aromatic hydrocarbons, including ethylbenzene, 4-xylene, and 1,3-bis-(1,1-dimethylethyl)benzene, warrants careful consideration. These compounds are not typical endogenous metabolites of Apiaceae seeds, which primarily biosynthesize terpenoids, phenylpropanoids, and fatty acid derivatives. The presence of these aromatic compounds likely reflects environmental contamination absorbed during seed development in the wild collection site (Tonghua County, Jilin Province) or introduced during post-harvest processing and storage. Solvent blank controls were analyzed to exclude extraction solvent impurities; however, complete elimination of the environmental background in field-collected samples is technically challenging. We explicitly state that these aromatic compounds are not considered biologically relevant to seed dormancy, and the dormancy-related inhibitory activity is attributed to the dominant polar constituents (acetic acid, palmitic acid, thymol) detected in the methanol phase.

Furthermore, the direct-injection GC-MS approach employed here, while effective for profiling volatile and semi-volatile compounds, inherently excludes polar metabolites and compounds with molecular weights exceeding approximately 500 Da. This methodological constraint means that potential dormancy-related compounds such as sugars, amino acids, and larger secondary metabolites were not captured in the present analysis. Future investigations integrating derivatization-GC-MS and LC-MS platforms will be necessary to achieve comprehensive metabolomic coverage of seed extracts during dormancy release. Due to the absence of bio-guided fractionation and equipment blank controls, this study does not claim that any single compound is solely responsible for the observed dormancy.

### 3.2. Complementary Metabolomic and Transcriptomic Analyses

While GC-MS profiling provides a static chemical snapshot of the dormant state, transcriptomic analysis reveals the dynamic gene regulatory networks underlying dormancy release. The integration of these complementary datasets offers a preliminary framework for understanding the metabolic–genetic axis of the physiological component of MPD in *S. brachycarpa.*

The GC-MS profiling identified phenolic compounds (hydroquinone, thymol) and organic acids (acetic acid, palmitic acid) as dominant constituents in the inhibitory methanol phase. Transcriptomic analysis further revealed that genes encoding phenylalanine ammonia-lyase (PAL) and lipoxygenase (LOX)—key enzymes in phenylpropanoid and oxylipin biosynthesis pathways—were significantly differentially expressed during stratification. Although the metabolomic and transcriptomic datasets were derived from different experimental contexts (static chemical profiling of freshly harvested seeds vs. dynamic gene expression during stratification-induced dormancy release), the convergence on phenylpropanoid and fatty acid metabolism pathways suggests these processes are central to the physiological component of MPD in *S. brachycarpa.*

Furthermore, the upregulation of genes involved in gibberellin (GA) biosynthesis and signaling, coupled with the downregulation of abscisic acid (ABA) pathway genes at 120 days of stratification, aligns with the established hormonal regulation of dormancy release. The co-occurrence of phenolic inhibitors in the dormant state and the transcriptional activation of hormone metabolism during stratification supports a model in which the physiological component of MPD is maintained by endogenous chemical inhibition and released through hormone-mediated transcriptional reprogramming.

### 3.3. Limitations and Future Directions

RIN values ranged from 5.00 to 8.20. Although two samples had an RIN around 5.0, all samples showed distinct 28S/18S peaks without obvious degradation smearing, and the sequencing quality metrics (Q20 ≥ 99.20%, Q30 ≥ 96.22%) were satisfactory. Nevertheless, we cannot fully exclude a minor effect of RNA integrity on expression estimates, and this limitation should be considered when interpreting the transcriptomic results.

It is important to emphasize that the associations between the identified compounds and germination inhibitory activity reported in this study are strictly correlative. In the absence of dose–response assays with purified compounds, fraction-specific bioassays, and direct testing on *S. brachycarpa* embryos, causality cannot be established. The bioassay results using Brassica rapa seeds provide evidence of inhibitory potential but do not demonstrate that these compounds are the causal agents of dormancy in the target species.

Furthermore, it should be noted that the metabolomic and transcriptomic analyses were conducted on different sample sets (freshly harvested seeds for GC-MS vs. stratified seeds for RNA-Seq), reflecting the complementary rather than directly comparative nature of these datasets. The metabolic profiling captures the chemical composition of the dormant state, whereas the transcriptomic profiling captures gene expression dynamics during dormancy release. Future studies employing simultaneous metabolomic and transcriptomic profiling of identical samples during stratification time courses will be necessary to establish direct metabolite–gene expression correlations and confirm the regulatory relationships proposed herein.

Additionally, the direct-injection GC-MS approach employed here inherently excludes polar metabolites and compounds with molecular weights exceeding approximately 500 Da. Derivatization-GC-MS or LC-MS platforms will be required for comprehensive metabolomic coverage in future investigations. Similarly, the transcriptomic analysis was limited to three time points (0, 60, and 120 days) selected based on germination phenotypes; finer temporal resolution may reveal additional regulatory nodes in the dormancy release network.

## 4. Materials and Methods

### 4.1. Experimental Design

This study integrates two complementary experimental approaches to investigate the morphophysiological dormancy (MPD) of *S. brachycarpa* seeds. For metabolic profiling, freshly harvested seeds (September 2022, Tonghua County, Jilin Province) were used without stratification to characterize endogenous inhibitor composition by GC-MS. For transcriptomic analysis, seeds from the same collection batch were subjected to cold stratification at 4 °C for 0, 20, 40, 60, 80, 100, and 120 days. Samples at 0, 60, and 120 days were selected for RNA sequencing based on germination phenotype monitoring: 0-day seeds exhibited near-zero germination (deep dormancy), 60-day seeds showed partial germination recovery (transitional dormancy release), and 120-day seeds achieved greater than 80% germination (full dormancy release), representing three biologically distinct stages of the dormancy-to-germination continuum. The static metabolic dataset captures the chemical composition of the dormant state, while the dynamic transcriptomic dataset reveals gene regulatory networks activated during dormancy release. The convergence of these complementary datasets provides multi-omics insights into the physiological component of MPD in *S. brachycarpa*.

The 0, 60, and 120 d time points were selected based on preliminary germination tests and previous reports on Apiaceae seed dormancy, representing the dormancy maintenance, mid-release, and germination-release stages, respectively. Germination was monitored weekly during stratification. Germination percentages were approximately 0% on day 0 (deep dormancy), 35–45% on day 60 (partial dormancy release), and >80% on day 120 (full dormancy release). These three stages represent the dormancy–germination continuum and were selected for transcriptomic sampling.

The heterologous bioassay was used only as a preliminary screening tool to rank the inhibitory potential of solvent fractions; it does not constitute causal evidence for dormancy regulation in *S. brachycarpa*. This study is, therefore, hypothesis-generating rather than mechanistic.

### 4.2. Material

*S. brachycarpa* seeds collected from the mountainous area of Tonghua County, Tonghua City, Jilin Province, in September 2022 were used as experimental materials. Seeds were selected using a 20-mesh sample sieve, and full seeds on the sieve were taken as experimental materials, which were dried in a cool and ventilated place indoors and then properly preserved. The thousand-seed weight was 5.53 g, and the longitudinal axis diameter was 2.63 mm, the diameter of transverse axis was 1.85 mm, and the seed thickness was 1.98 mm. The experiment was carried out in the protected horticulture laboratory of Jilin Agricultural University. Chinese cabbage seeds were purchased from Qingdao Jiaoyan Seedling Co., Ltd. (Qingdao, China), with seed purity ≥ 96%, clarity ≥ 98%, germination percentage ≥ 85% and water content of 7%. These seeds were selected as a positive control due to their rapid and uniform germination characteristics and their widespread use as a reference material in seed physiology studies.

Following collection, seeds were air-dried indoors under shade at ambient temperature (15–20 °C) for two weeks to a moisture content suitable for storage, then sealed and stored at 4 °C until use. All seeds used for metabolic profiling were freshly harvested material that had not undergone stratification.

### 4.3. Extraction of Endogenous Compounds

Seeds (5 g) were ground in liquid nitrogen and extracted three times with 25 mL of 80% (*v*/*v*) methanol, diethyl ether, petroleum ether, or ethyl acetate, respectively. Each extraction was performed at 4 °C for 24 h with agitation every 6 h. After each extraction, the slurry was filtered, and the filtrates from the three successive extractions were combined. The combined filtrate was concentrated to 5 mL via rotary evaporation at 45 °C, yielding extracts equivalent to 1 g seed fresh weight per mL, and stored at 4 °C until analysis [27,28].

Plump Chinese cabbage seeds were soaked in warm water at 45 °C for 30 min, then fished out and placed in a petri dish with a diameter of 10 cm and a double-layer moist filter paper, with 100 seeds in each dish. In petri dishes, 5 mL of extracts was put into each dish, 5 mL of distilled water was used as control, and each treatment was repeated 3 times. All petri dishes were cultured in a constant-temperature light incubator (25 °C, 12 h illumination/12 h darkness). Afte 48 h, the germination percentage of Chinese cabbage seeds was counted (cotyledons exposed as the germination standard). After 72 h, the root length and seedling length of Chinese cabbage seeds were measured with vernier caliper.

### 4.4. Bioassay with Chinese Cabbage Seeds

Chinese cabbage seeds were surface-treated by soaking in warm water (45 °C) for 30 min, then placed on two layers of moist filter paper in 10 cm petri dishes (100 seeds per dish). Thus, 5 mL of each extract was added per dish; distilled water served as the control, and a solvent blank (water extract prepared identically) was included to exclude nonspecific effects. Each treatment comprised three biological replicates. Petri dishes were incubated at 25 °C under a 12 h light/12 h dark photoperiod. Germination (radicle emergence) was recorded at 48 h; root length and shoot length were measured with a vernier caliper at 72 h.

Compound identification was performed by comparing the mass spectra with the NIST 2017 Standard Reference Database. The similarity threshold was set at >80%, and the retention time offset for identified compounds was required to be <1 min across samples. It should be noted that direct-injection GC-MS without derivatization was employed in this study, which limits detection to volatile and semi-volatile compounds with molecular weights typically below 500 Da. Polar compounds (e.g., sugars, amino acids) and high-molecular-weight substances are not detected under these conditions. Derivatization-GC-MS or LC-MS would be required for comprehensive metabolomic profiling of polar and high-molecular-weight metabolites in future studies.

Data processing was conducted using LECO ChromaTOF v5.0 software. The relative peak area (RPA) of each compound was calculated as the percentage of its peak area relative to the total peak area of all detected compounds in the chromatogram. All compound identifications reported in this study are putative assignments based on NIST/Wiley library spectral matching (match scores > 80%) and retention time consistency. Due to the absence of authentic reference standards and high-resolution mass spectrometry in our laboratory, these identities require confirmation through future validation using standard compounds or HRMS analysis.

Solvent blank controls were prepared by subjecting pure methanol, diethyl ether, petroleum ether, and ethyl acetate to identical extraction and concentration procedures without seeds. These controls were analyzed by GC-MS to exclude potential interference from solvent impurities.

### 4.5. Bioassay Using Chinese Cabbage Seeds

Chinese cabbage (*Brassica rapa* subsp. *pekinensis*) seeds were purchased commercially as a standard bioassay material to evaluate the inhibitory effects of *S. brachycarpa* seed extracts. This species was selected because (1) it exhibits rapid and uniform germination under standard conditions (non-dormant), allowing sensitive detection of germination inhibition; (2) it is genetically stable and not genetically modified; and (3) it has been widely used in previous studies for assessing allelopathic and inhibitory effects of plant extracts. The use of a non-target species as a bioindicator is a standard approach in seed biology to isolate and quantify the inhibitory potential of endogenous compounds without confounding effects of species-specific dormancy mechanisms.

Aqueous extraction was not included because prior work on Apiaceae seed dormancy has shown that the principal endogenous germination inhibitors are organic-soluble compounds (fatty acids, phenolics, terpenoids), whereas water-soluble fractions (e.g., sugars, amino acids) are generally promotive or neutral. Nonetheless, we acknowledge that excluding aqueous extracts may overlook hydrophilic inhibitory compounds, which will be examined in future work using LC-MS-based profiling.

Bioassays with a heterologous indicator species were employed as a preliminary screening tool to rank the inhibitory potential of solvent fractions; they do not constitute causal evidence for dormancy in *S. brachycarpa* itself.

### 4.6. Data Analysis

Microsoft Excel 2016 was used to collate the data and draw charts, and SPSS 26.0 software was used to analyze the difference significance of the experimental data. Prior to analysis, data were checked for normality using the Shapiro–Wilk test and for homogeneity of variance using Levene’s test. One-way ANOVA was conducted to compare means among treatments, followed by Duncan’s multiple range test (DMRT) for post hoc comparisons when significant differences were detected. Statistical significance was set at *p* < 0.05. All data are presented as mean ± standard error (SE).

### 4.7. RNA Extraction, Library Construction, and Sequencing

Total RNA was extracted from *S. brachycarpa* seeds using TRIzol reagent (Thermo Fisher, cat. 15596018) according to the manufacturer’s instructions. RNA quantity and purity were assessed with a NanoDrop ND-1000 spectrophotometer (NanoDrop, Wilmington, DE, USA), and RNA integrity was evaluated using a Bioanalyzer 2100 (Agilent, Santa Clara, CA, USA). Samples with concentration > 50 ng/μL, RIN > 5.0, and total RNA > 1 μg were used for library construction.

Poly(A)-containing mRNA was enriched using oligo(dT) magnetic beads (Dynabeads Oligo(dT), cat. 25-61005, Thermo Fisher, Waltham, MA, USA) through two rounds of purification. The captured mRNA was fragmented using the NEBNext Magnesium RNA Fragmentation Module (cat. E6150S, NEB, Ipswich, MA, USA) at 94 °C for 5–7 min. First-strand cDNA was synthesized using Invitrogen SuperScript II Reverse Transcriptase (cat. 1896649, CA, USA). Second-strand synthesis was performed with *E. coli* DNA polymerase I (NEB, cat. m0209, USA) and RNase H (NEB, cat. m0297, Ipswich, MA, USA), with dUTP Solution (Thermo Fisher, cat. R0133, CA, USA) incorporated to generate blunt-ended double-stranded DNA. An A base was added to the 3′ ends, followed by ligation with T-base adapters, and fragments were size-selected and purified with magnetic beads. The second strand was digested with UDG enzyme (NEB, cat. m0280, Ipswich, MA, USA), followed by PCR amplification (pre-denaturation at 95 °C for 3 min; 8 cycles of 98 °C for 15 s, 60 °C for 15 s, 72 °C for 30 s; final extension at 72 °C for 5 min) to generate a strand-specific library with an insert size of 300 bp ± 50 bp. Sequencing was performed on the Illumina NovaSeq 6000 platform (LC Bio Technology Co., Ltd., Hangzhou, China) in paired-end 150 bp (PE150) mode.

### 4.8. Bioinformatics Analysis

Raw reads were trimmed with fqtrim to remove adapter sequences and low-quality bases. Clean reads were de novo assembled using Trinity with default parameters. Transcript abundance was quantified as TPM using RSEM after mapping clean reads back to the assembled transcripts with Bowtie2.

## Figures and Tables

**Table 1:** Effect of extracts of each organic phase of *Spuriopinella brachycarpa* (Kom.) Kitag seeds on the germination and growth of cabbage seeds (the lowercase letters in the same column in the table indicate the multiple comparison of germination percentage of Chinese cabbage seeds at 0.05 level under different extract treatments).

Organic Solvents	Germination Percentage (%)	Root Length (cm)	Seedling Height (cm)
Methanol phase	13.67 ± 1.53 c	1.37 ± 0.12 d	1.60 ± 0.10 d
Ether phase	45.00 ± 1.00 b	1.83 ± 0.06 c	2.27 ± 0.06 c
Petroleum ether phase	79.33 ± 2.31 a	2.97 ± 0.25 b	3.47 ± 0.15 b
Ethyl acetate phase	78.00 ± 3.61 a	2.83 ± 0.25 b	3.60 ± 0.10 ab
Control	82.33 ± 2.08 a	3.60 ± 0.36 a	3.93 ± 0.49 a

**Table 2:** Main chemical components and relative signal intensity (RPA) of the methanol-phase extracts of the concentrated samples of *Spuriopinella brachycarpa* (Kom.) Kitag seeds (“-” in the table means that the substance does not match the specific classification information in the HMDB database, the same below).

Retention Time	Molecular Formula	Molecular Weight	Substance Name	Substance Classification	Relative Content
41.72	C_4_H_6_O_3_	102.09	3, 4-dihydroxybutanoic acid gamma-lactone	-	0.22%
33.24	C_10_H_16_O	152.23	4-isopropenyl-1-methyl- cyclohex-2-en-1-ol, (1r*, 4r*), rel-		1.02%
4.39	C_2_H_6_O	46.07	Ethanol	Organic oxygen compound	0.67%
9.17	C_8_H_10_	106.16	Ethylbenzene	Benzene	0.19%
9.50	C_8_H_10_	106.16	4-xylene	Benzene	3.56%
9.66	C_5_H_7_N	81.12	n-methylpyrrole	Pyrrolidone	6.34%
13.95	C_5_H_6_N_2_	94.11	2-methylpyrazine	Pyrazines	0.26%
14.96	C_3_H_6_O_2_	74.08	Acetol	Organic oxygen compound	4.51%
15.59	C_6_H_8_N_2_	108.14	2, 5-dimethylpyrazine	Pyrazines	0.27%
17.88	C_7_H_10_N_2_	122.17	2, 3, 5-trimethylpyrazine	Pyrazines	0.33%
19.01	C_2_H_4_O_2_	60.05	acetic acid	Organic acids	9.00%
22.41	C_12_H_20_O_2_	196.29	Monoterpene derivative	-	6.91%
23.32	C_4_H_6_O_2_	86.09	Butyrolactone	Lactones	0.63%
29.33	C_2_H_6_O_2_S	94.14	dimethyl sulfone	Organic sulfide	0.01%
32.26	C_4_H_7_NO	85.1	2-pyrrolidone	Pyrrolidone	0.72%
33.02	C_3_H_6_O_3_	90.08	Dihydroxyacetone	Organic oxygen compound	0.27%
34.61	C_4_H_6_O_3_	102.09	hydroxy-gamma-butyrolactone	Lactones	0.26%
45.20	C_16_H_32_O_2_	256.42	Palmitic acid	Fatty acids	1.50%
46.76	C_6_H_6_O_2_	110.11	Hydroquinone	Phenols	1.29%
46.80	C_17_H_24_O	244.37	1,9-heptadecadiene-4,6-diyn-3-ol, (3s, 9z)-	-	1.31%
48.69	C_4_H_6_N_2_O_2_	114.1	5-methylhydantoin	-	0.25%
30.19	C_3_H_5_NO	71.08	Acrylamide	-	0.25%

**Table 3:** The types and relative signal intensity (RPA) of organic compounds extracted by ether phase from the seed of *Spuriopinella brachycarpa* (Kom.) Kitag seeds.

Retention Time	Molecular Formula	Molecular Weight	Substance Name	Substance Classification	Relative Signal Intensity (RPA)
29.62	C_15_H_24_O	220.35	Butylated Hydroxytoluene	Phenols	1.29%
22.10	-	-	acetic acid--(1s, 5r, 6r)-2,7,7-trimethylbicyclo [3.1.1] hept-2-en-6-ol (1/1)	-	1.87%
6.00	C_10_H_16_	136.23	Alpha-pinene	Monoterpene	0.29%
7.30	C_10_H_16_	136.23	Camphene	Monoterpene	0.52%
9.13	C_10_H_16_	136.23	Sabinene	Monoterpene	0.30%
10.63	C_10_H_16_	136.23	MyrCene	Monoterpene	1.05%
11.61	C_10_H_16_	136.23	limonene	Monoterpene	3.08%
11.86	C_10_H_16_	136.23	Beta-phellandrene	Monoterpene	1.72%
14.22	C_10_H_16_	136.23	Terpinolene	Monoterpene	0.08%
18.37	C_10_H_16_	136.23	Benzene, 1,3-bis (1,1-dimethylethyl)-	Monoterpene	0.44%
20.73	C_7_H_6_O	106.12	Benzaldehyde	Benzene	0.32%
22.41	C_12_H_20_O_2_	196.29	Monoterpene derivative	-	13.84%
22.72	C_15_H_24_	204.36	Sesquiterpene	Sesquiterpenes	1.55%
45.20	C_16_H_32_O_2_	256.42	Palmitic acid	Fatty acids	1.49%
47.26	C_30_H_50_	410.73	Triterpene	-	0.20%

**Table 4:** The types of organic compounds extracted from the seeds of *Spuriopinella brachycarpa* (Kom.) Kitag using the petroleum ether phase, along with their relative signal intensity (RPA).

Retention Time	Molecular Formula	Molecular Weight	Substance Name	Substance Classification	Relative Content
18.37	C_10_H_16_	136.23	Benzene, 1,3-bis (1,1-dimethylethyl)-	Monoterpene	0.41%
6.00	C_10_H_16_	136.23	Alpha-pinene	Monoterpene	1.76%
6.18	C_10_H_16_	136.23	beta-thujene, (+/−)-	Monoterpene	0.25%
7.30	C_10_H_16_	136.23	Camphene	Monoterpene	2.27%
8.60	C_10_H_16_	136.23	(-)-beta-pinene	Monoterpene	0.77%
9.13	C_10_H_16_	136.23	Sabinene	Monoterpene	1.36%
10.63	C_10_H_16_	136.23	Myrcene	Monoterpene	3.19%
13.28	C_10_H_16_	136.23	gamma-terpinene	Monoterpenes	13.90%
14.26	C_14_H_30_	198.39	4,6-dimethyldodecane	-	0.61%
20.73	C_7_H_6_O	106.12	Benzaldehyde	Benzene	0.55%
22.72	C_15_H_24_	204.35	Sesquiterpene	Sesquiterpenes	1.85%
25.24	C_15_H_24_	204.35	gamma-cadinene	Sesquiterpene	0.17%
49.04	C_18_H_34_O_2_	282.5	cis-vacenic acid	Fatty acids	1.32%
14.26	C_14_H_30_	198.39	4,6-dimethyldodecane	-	0.61%
22.72	C_15_H_24_	204.35	beta-caryophyllene	Sesamines	1.85%

Note: “-” indicates that the substance does not match specific classification information in the HMDB database.

**Table 5:** Species and relative signal intensity (RPA) of organic compounds in the ethyl acetate-phase extract of *Spuriopinella brachycarpa* (Kom.) Kitag. seeds.

Retention Time	Molecular Formula	Molecular Weight	Substance Name	Substance Classification	Relative Content
6.00	C_10_H_16_	136.23	Alpha-pinene	Monoterpene	0.21%
7.30	C_10_H_16_	136.23	Camphene	Monoterpene	0.31%
9.13	C_10_H_16_	136.23	Sabinene	Monoterpene	0.12%
10.63	C_10_H_16_	136.23	MyrCene	Monoterpene	0.70%
11.61	C_10_H_16_	136.23	Limonene	Monoterpene	0.88%
11.86	C_10_H_16_	136.23	Beta-phellandrene	Monoterpene	1.49%
22.41	C_12_H_20_O_2_	196.29	bornyl acetate	-	8.89%
47.26	C_30_H_50_	410.72	Squalene	-	0.19%
45.20	C_16_H_32_O_2_	256.42	Palmitic acid	Fatty acids	0.60%
19.00	C_2_H_4_O	60.05	acetic acid	Organic Acids	1.27%
22.72	C_15_H_24_	204.35	beta-caryophyllene	Sesamines	1.49%

Note: “-” indicates that the substance does not match specific classification information in the HMDB database.

**Table 6:** Screening table for organic compounds in extracts of each organic phase of extracts from *Spuriopinella brachycarpa* (Kom.) Kitag seeds (note: “-” indicates that the relative signal intensity (RPA) of the substance is less than 0.01%).

Name in Chinese	Substance Name	Substance Classification	Relative Content of Each Substance			
			Methanol Phase	Ether Phase	Petroleum Ether Phase	Ethyl Acetate Phase
n-methylpyrrole	n-Methylpyrrole	Pyrrolidone	6.34%	-	-	-
acetol	Acetol	Organic oxide	4.51%	-	-	-
acetic acid	Acetic acid	Organic acid	9.00%	-	-	1.27%
2-hydroxy-gamma-butyrolactone	2-Hydroxy-γ-butyrolactone	-	2.79%	-	-	-
benzaldehyde	Benzaldehyde	Benzene	-	0.32%	0.55%	-
hydroquinone	Hydroquinone	Phenol	1.29%	-	-	-
Butylated Hydroxytoluene	Butylated hydroxytoluene	Benzene	-	1.29%	0.01%	-
4-isopropenyl-1-methyl-cyclohex-2-en-1-ol, (1r*, 4r*), rel-	4-Isopropenyl-1-methylcyclohex-2-en-1-ol	Monoterpene	1.02%	-	-	0.06%
1,9-heptadecadiene-4,6-diyn-3-ol, (3s, 9z)-	1,9-Heptadecadiene-4,6-diyn-3-ol	-	1.31%	0.40%	0.55%	0.65%
palmitic acid	Palmitic acid	Fatty acid	1.50%	1.49%	0.59%	0.60%
n-dodecane	n-Dodecane	Alkane	0.14%	-	0.06%	0.01%
ethylbenzene	Ethylbenzene	Ethylbenzene	1.83%	-	-	-
4-xylene	4-Xylene	4-Xylene	3.56%	0.01%	0.01%	-
thymol	Thymol	Monoterpene	1.89%	0.01%	0.01%	-

**Table 7:** Transcriptome data quality summary of *Spuriopinella brachycarpa* (Kom.) Kitag. seeds.

Sample	Raw_Reads	Raw_Bases	Valid_Reads	Vaild_Bases	Valids%	Q20%	Q30%	GC%	RIN
Dormancy1	41,124,876	6.17 G	40,314,028	5.97 G	98.03%	99.42	97.60	48.85	5.20
Dormancy2	46,658,656	7.00 G	45,666,490	6.76 G	97.87%	99.43	97.57	52.79	8.00
Dormancy3	35,877,932	5.38 G	35,057,054	5.18 G	97.71%	99.43	97.68	46.14	6.70
Mid1	49,610,280	7.44 G	48,828,442	7.24 G	98.42%	99.41	97.55	49.54	7.00
Mid2	38,310,060	5.75 G	37,506,932	5.55 G	97.90%	99.39	97.48	51.82	5.00
Mid3	43,131,840	6.47 G	42,285,064	6.26 G	98.04%	99.46	97.75	46.33	8.20
Release1	40,062,030	6.01 G	39,325,658	5.82 G	98.16%	99.40	97.48	52.51	7.10
Release2	42,716,008	6.41 G	42,009,970	6.23 G	98.35%	99.43	97.62	50.74	7.40
Release3	48,565,608	7.28 G	46,676,476	6.88 G	96.11%	99.20	96.22	51.30	7.60

Note: Sample, sample name; Raw_Reads, number of raw sequencing reads; Raw_Bases, raw data volume; Valid_Reads, number of valid reads; Valid_Bases, valid data volume; Valids%, proportion of valid reads; Q20%, proportion of bases with quality value ≥ 20 (sequencing error rate < 0.01); Q30%, proportion of bases with quality value ≥ 30 (sequencing error rate < 0.001); GC%, proportion of GC content; RIN, RNA Integrity Number, determined by Agilent Bioanalyzer 2100.

**Table 8:** Gene assembly summary of *Spuriopinella brachycarpa* (Kom.) Kitag. seeds.

Index	All	GC%	Min Length	Median Length	Max Length	Total Assembled Bases	N50
Transcript	307,034	48.96	176	869.00	23,082	353,290,335	1673
Gene	109,718	49.86	201	479.00	23,082	95,556,372	1506

**Table 9:** Gene function annotation summary of *Spuriopinella brachycarpa* (Kom.) Kitag. seeds.

	TF	NR	eggNOG	Swissport	Pfam	KEGG	GO	All
Number of Unigenes	1513	65,392	78,481	55,938	67,736	23,606	68,592	109,718
Percentage (%)	1.38	59.60	71.53	50.98	61.74	21.52	62.52	100.00

**Table 10:** Differentially expressed genes (DEGs) associated with hormone signal transduction during seed germination of *Spuriopinella brachyara* (Kom.) Kitag.

Gene ID	Gene Annotation	DvsM		DvsR	
		Log_2_ FC	*p*-Adjust	Log_2_FC	*p*-Adjust
TRINITY_DN1185_c0_g1	hypothetical protein *FH972_023474* [*Carpinus fangiana*]	+7.71	*p* < 0.05	+5.00	*p* < 0.05
TRINITY_DN21355_c0_g1	predicied abscisic acid receptor *PYL4*-like [*Daucus carota* subsp. sativus]			−3.22	*p* < 0.05
TRINITY_DN68528_c1_g1	hypothetical protein FH972_022520 [*Carpinus fangiana*]	+6.66	*p* < 0.05	+6.27	*p* < 0.05
TRINITY_DN12308_c0_g1	hypothetical protein *MRB53_040292* [*Persea americana*]	−10.82	*p* < 0.05		
TRINITY_DN21468_c0_g1	predicted protein [*Hordeum vulgare* subsp. *vulgare*]	+5.39	*p* < 0.05		
TRINITY_DN41542_c0_g2	Predicied *SCARECROW*-like protein 28 [*Daucus carota* subsp. *sativus*]	+2.96	*p* < 0.05		

## Data Availability

The original contributions presented in this study are included in the article. Further inquiries can be directed to the corresponding author(s).
